# Long COVID: Mechanismen, Risikofaktoren und Genesung

**DOI:** 10.1159/000529939

**Published:** 2023-03-20

**Authors:** Rónan Astin, Amitava Banerjee, Mark R. Baker, Melanie Dani, Elizabeth Ford, James H. Hull, Phang Boon Lim, Melitta McNarry, Karl Morten, Oliver O'Sullivan, Etheresia Pretorius, Betty Raman, Demetris S. Soteropoulos, Maxime Taquet, Catherine N. Hall

**Affiliations:** ^a^Department of Respiratory Medicine, University College London Hospitals NHS Foundation Trust, London, Vereinigtes Königreich; ^b^Centre for Human Health and Performance, Institute for Sport Exercise and Health, University College London, London, Vereinigtes Königreich; ^c^Institute of Health Informatics, University College London, London, Vereinigtes Königreich; ^d^Department of Cardiology, Barts Health NHS Trust, London, Vereinigtes Königreich; ^e^Faculty of Medical Sciences, Newcastle University, Newcastle upon Tyne, Vereinigtes Königreich; ^f^Imperial Syncope Unit, Imperial College Healthcare NHS Trust, London, Vereinigtes Königreich; ^g^Brighton and Sussex Medical School, Falmer, Vereinigtes Königreich; ^h^Institute of Sport, Exercise and Health (ISEH), Division of Surgery and Interventional Science, University College London, London, Vereinigtes Königreich; ^i^Royal Brompton Hospital, London, Vereinigtes Königreich; ^j^Applied Sports, Technology, Exercise and Medicine Research Centre, Swansea University, Swansea, Vereinigtes Königreich; ^k^Nuffield Department of Women's and Reproductive Health, University of Oxford, Oxford, Vereinigtes Königreich; ^l^Academic Department of Military Rehabilitation, Defence Medical Rehabilitation Centre Stanford Hall, Loughborough, Vereinigtes Königreich; ^m^School of Medicine, University of Nottingham, Nottingham, Vereinigtes Königreich; ^n^Department of Physiological Sciences, Faculty of Science, Stellenbosch University, Stellenbosch, Südafrika; ^o^Department of Biochemistry and Systems Biology, Institute of Systems, Molecular and Integrative Biology, Faculty of Health and Life Sciences, University of Liverpool, Liverpool, Vereinigtes Königreich; ^p^Radcliffe Department of Medicine, Division of Cardiovascular Medicine, University of Oxford, Oxford, Vereinigtes Königreich; ^q^Radcliffe Department of Medicine, Division of Cardiovascular Medicine, Oxford University Hospitals NHS Foundation Trust, Oxford, Vereinigtes Königreich; ^r^Department of Psychiatry, University of Oxford, Oxford, Vereinigtes Königreich; ^s^Oxford Health NHS Foundation Trust, Oxford, Vereinigtes Königreich; ^t^School of Psychology and Sussex Neuroscience, University of Sussex, Falmer, Vereinigtes Königreich

**Keywords:** Herz-Kreislauf, Gerinnung, Dysautonomie, Erschöpfung, Long COVID, ME/CFS, Atemwege, SARS-CoV-2

## Abstract

Long COVID, die lang anhaltende Krankheit und Erschöpfung, die bei einem kleinen Teil der SARS-CoV-2-Infizierten auftritt, stellt eine zunehmende Belastung für die Betroffenen und die Gesellschaft dar. Eine virtuelle Tagung der Physiological Society im Februar 2022 brachte Kliniker und Forscher zusammen, um das aktuelle Verständnis der Mechanismen, Risikofaktoren und Genesung nach Long COVID zu erörtern. In dieser Übersichtsarbeit werden die Themen behandelt, die sich aus dieser Tagung ergeben haben. Die Übersichtsarbeit befasst sich mit der Natur von Long COVID, untersucht den Zusammenhang mit anderen postviralen Erkrankungen wie der myalgischen Enzephalomyelitis/dem chronischen Erschöpfungssyndrom und zeigt auf, wie die Forschung zu Long COVID helfen kann, Patienten mit allen möglichen postviralen Syndromen besser zu unterstützen. Die Forschung zu Long COVID hat besonders rasche Fortschritte bei Bevölkerungsgruppen gemacht, die ihre körperliche Leistungsfähigkeit routinemäßig überwachen, insbesondere beim Militär und bei Leistungssportlern. In der Übersichtsarbeit wird hervorgehoben, inwiefern das hohe Niveau von Diagnose, Intervention und Erfolgskontrolle in diesen aktiven Bevölkerungsgruppen Informationen über Managementstrategien für die Allgemeinbevölkerung liefern kann. Anschließend wird untersucht, wie eine Schlüsselkomponente der Leistungsüberwachung bei diesen aktiven Bevölkerungsgruppen, das kardiopulmonale Training, Long-COVID-bedingte Veränderungen in der Physiologie aufdeckt − einschließlich Veränderungen der peripheren Muskelfunktion, der ventilatorischen Ineffizienz und der autonomen Dysfunktion. Das Wesen und die Auswirkungen der Dysautonomie werden im Zusammenhang mit dem posturalen orthostatischen Tachykardiesyndrom, der Fatigue und den Behandlungsstrategien, die darauf abzielen, der Überaktivierung des Sympathikus durch Stimulation des Vagusnervs entgegenzuwirken, erörtert. Anschließend untersuchen wir die Mechanismen, die den Symptomen von Long COVID zugrunde liegen. Dabei konzentrieren wir uns auf die gestörte Sauerstoffversorgung durch Mikrokoagulation und die Störung des zellulären Energiestoffwechsels, bevor wir Behandlungsstrategien betrachten, die direkt oder indirekt auf diese Mechanismen abzielen. Dazu gehören ein fernbetreutes Atemmuskeltraining und integrierte Versorgungspfade, die Rehabilitation und medikamentöse Interventionen mit der Erforschung des Zugangs zur Long-COVID-Versorgung in verschiedenen Bevölkerungsgruppen kombinieren. Insgesamt zeigt diese Übersichtsarbeit, wie im Rahmen der physiologischen Forschung die bei Long COVID auftretenden Veränderungen aufgedeckt werden und wie verschiedene therapeutische Strategien zur Bekämpfung dieser Erkrankung entwickelt und getestet werden.

## Neue Erkenntnisse

• Was ist das Thema dieser Übersichtsarbeit?
- Das neue Krankheitsbild von Long COVID, die Epidemiologie, die pathophysiologischen Auswirkungen auf Patienten mit unterschiedlichem Hintergrund, die physiologischen Mechanismen, die sich als Erklärung für das Krankheitsbild abzeichnen, und die erprobten Behandlungsstrategien. Die Übersichtsarbeit ist das Ergebnis einer Online-Konferenz der Physiological Society zu diesem Thema.
• Welche Fortschritte werden hervorgehoben?
- Fortschritte im Verständnis der Pathophysiologie und der zellulären Mechanismen, die Long COVID zugrunde liegen, sowie potenzielle Therapie- und Managementstrategien.

## Einleitung

Long COVID, d.h. 12 Wochen oder länger anhaltende Symptome nach einer Coronavirus-Erkrankung (coronavirus disease, COVID), bedrohen Einzelpersonen, Bevölkerungsgruppen und Volkswirtschaften. Allein in Großbritannien sind schätzungsweise 14, Millionen Menschen betroffen, weltweit 144 Millionen [1, 2]. Das Problem der postviralen Syndrome ist nicht neu, aber das Ausmaß und die Geschwindigkeit dieser globalen Herausforderung erfordern ein vernetztes Denken über die traditionellen klinischen und akademischen Grenzen hinweg. Da es sich bei Long COVID um eine neue Erkrankung handelt, müssen ihre Epidemiologie, Risikofaktoren und Mechanismen identifiziert werden, um die Ätiologie zu verstehen, die Diagnose zu erleichtern und neue Behandlungsstrategien zu entwickeln. Neue Forschungsarbeiten und fortgesetzte Untersuchungen und Behandlungsversuche sind unerlässlich, um eine Erkrankung mit solch schwerwiegenden Auswirkungen auf die Gesundheit der Bevölkerung und der einzelnen Betroffenen zu bekämpfen. Diese Arbeit spiegelt viele Perspektiven einer kürzlich stattgefundenen Tagung der Physiological Society («Long COVID: Mechanisms, Risk Factors and Recovery») wider, bei der verschiedene Forschungsgruppen, die sich mit Long COVID beschäftigen, zusammenkamen, um ein besseres Verständnis der Krankheit zu erlangen und Richtungen für die zukünftige Forschung und Behandlung aufzuzeigen.

## Die Bedeutung von Daten bei der Klassifizierung von Long COVID

Es ist von entscheidender Bedeutung, dass Long COVID konsistent als Krankheit beschrieben wird und die Prävalenzraten korrekt erhoben werden, aber beides ist nicht einfach. Das Design einer Long-COVID-Studie und die Rekrutierung können die Einschätzung der Prävalenz, der berichteten Symptome und ihrer Dauer sowie der damit verbundenen Risikofaktoren erheblich beeinflussen. Weitere zu berücksichtigende Faktoren sind der Teststatus, die Präsentation und die Dauer. Die Messung der Prävalenz und Inzidenz von Long COVID ist daher schwierig, aber unerlässlich, wenn die Auswirkungen auf die Betroffenen und die Gesellschaft angemessen berücksichtigt werden sollen. In den ersten Tagen der Pandemie hatten viele Menschen mit COVID-19 keinen Zugang zu COVID-19-Tests und konnten nicht für Studien rekrutiert werden. Aus diesem Grund wurden Long-COVID-Raten zunächst für Krankenhauspatienten geschätzt, bei denen nach der intensivmedizinischen Behandlung Krankheiten und Behinderungen zu erwarten waren (bis zu 87%) [3]. Später kam es auf Druck der Patienten dazu, dass Long COVID auch in nicht hospitalisierten Bevölkerungsgruppen anerkannt wurde [4]. Die Diagnose anhand von Symptomclustern war eine Herausforderung, da Long-COVID-Symptome (Erschöpfung, Kopfschmerzen, Gehirnnebel, Muskel- und Skelettschmerzen, Atembeschwerden, Brustschmerzen) in der Allgemeinbevölkerung weit verbreitet sind [5]. Daher können unkontrollierte Studien nicht ohne weiteres zwischen dem Ausmaß und der Schwere der Symptome von Personen mit und ohne Exposition gegenüber COVID-19 unterscheiden. Hinzu kommen Pandemiemaßnahmen wie Lockdowns und Schulschließungen, die bei vielen Menschen zu erhöhtem Stress, sozialer Isolation, veränderten Lebensgewohnheiten, verminderter körperlicher Aktivität, Beziehungsschwierigkeiten sowie sozialer und wirtschaftlicher Unsicherheit führten, die unabhängig voneinander mit verstärkten körperlichen Symptomen einhergingen [5]. Ferner ist es nicht möglich, die Wirkung von COVID-19 auf bereits bestehende Symptome zu bestimmen, da keine Daten über die Ausgangssituation vor der Erkrankung vorliegen. Es gibt keine einheitliche Definition der Symptomdauer. Die Studien messen zwischen 3 und 24 Wochen, eine Heterogenität, die Meta-Analysen ausschließt [6]. Weitere Komplikationen sind der unterschiedliche Impfstatus und die im Laufe der Zeit vorherrschende COVID-19-Variante. Studien, für die Patienten mit selbstberichtetem Long COVID rekrutiert wurden, können Merkmale und Auswirkungen [7], Organschäden und physiologische Veränderungen [8] beschreiben, aber aufgrund des Bias durch Selbstselektion weder Prävalenz noch Dauer schätzen. Retrospektive Kohorten (Post-COVID-19-Infektion) helfen bei der Schätzung der Prävalenz, können aber die Auswirkungen von COVID-19 auf chronische Symptome überschätzen, wenn keine Basis- oder Vergleichsgruppen vorhanden sind [9].

Das beste Studiendesign, um die Prävalenz und die Dauer von Long COVID zu verstehen, ist eine prospektive Kohorte, idealerweise mit Ausgangsdaten vor der Erkrankung und einer äquivalenten nicht exponierten Vergleichsgruppe. Die App «COVID-19 Symptom Tracker» bietet diese Möglichkeit [10] − Nutzer zeichnen ihre Daten in der Regel vor einer COVID-19-Infektion auf und können im Falle einer Infektion mit ähnlichen Nutzern verglichen werden, die nicht an COVID-19 erkrankt sind. Andere Kohortenquellen sind die UK Biobank [11] oder bevölkerungsbezogene elektronische Patientenakten [12], wobei Letztere jedoch dadurch eingeschränkt sind, dass sie die Konsultation und korrekte Kodierung durch einen Angehörigen der Gesundheitsberufe erfordern.

## Prävalenz, Eigenart und Unterschiede zu anderen postviralen Erkrankungen

Angesichts der oben genannten Herausforderungen können re­trospektive Kohortenstudien, die auf routinemäßig erhobenen Daten aus elektronischen Patientenakten (electronic health records, EHR) basieren, hilfreich sein. Solche Studien beruhen nicht auf der Selbstangabe von Symptomen durch Patienten oder auf Selbstrekrutierung. Sie können auch Ausgangsdaten für jeden Patienten und Kontrollgruppen bereitstellen.

EHR-Daten von mehr als 81 Millionen Menschen, hauptsächlich in den USA, einschließlich 273 618 bestätigter COVID-19-Patienten, wurden zur Schätzung der Inzidenz von 9 Long-COVID-Merkmalen 3–6 Monate nach COVID-19 im Vergleich zur Influenza herangezogen [13]. Die Inzidenz der einzelnen Merkmale in der COVID-19-Kohorte betrug 42,34%, wobei die Prävalenz jedes der Symptome in Tabelle 1 dargestellt ist. Alle 9 Merkmale traten nach COVID-19 häufiger auf als nach Influenza (insgesamt 16,60%, Hazard Ratio 1,44–2,04, alle *P* < 0,001), wobei eine Koexistenz von Symptomen deutlich wahrscheinlicher war als nach Influenza. Wenn Long-COVID-Merkmale als Netzwerk von Symptomen dargestellt werden (jeder Knoten ist ein Symptom, jede Kante steht für die Wahrscheinlichkeit ihres gemeinsamen Auftretens), zeigt sich, dass dieses Netzwerk sich mit zunehmender Zeit nach der COVID-19-Infektion verdichtet. Mit anderen Worten: Wenn ein postakutes Symptom aufgezeichnet wurde, trat es mit größerer Wahrscheinlichkeit in einer Konstellation mit anderen Symptomen auf (im Vergleich zur akuten Phase). Bemerkenswert ist, dass bei 30% der Personen, die an einer Influenza erkrankt waren, 3 bis 6 Monate nach der Infektion mindestens 1 Merkmal festgestellt wurde. Dies war zwar deutlich weniger als bei COVID-19, aber die Inzidenz ist nicht unerheblich und könnte darauf hindeuten, dass ein Teil der Belastung durch Long COVID auf eine allgemeine Erkrankung nach der Infektion zurückzuführen ist.

Unterschiede in den Long-COVID-Merkmalen wurden auch zwischen verschiedenen Untergruppen von Patienten festgestellt. Weibliche Patienten litten häufiger an Kopfschmerzen, Myalgien und abdominalen Symptomen und seltener an Atembeschwerden und kognitiven Defiziten als männliche Patienten. Krankenhauspatienten und ältere Menschen litten häufiger an kognitiven Problemen, Atembeschwerden und Erschöpfung und seltener an Kopfschmerzen und Myalgien als nicht hospitalisierte oder jüngere Patienten.

Eine andere Studie derselben Forschungsgruppe, die dieselbe EHR-Methodik verwendete [14], zeigte, dass das Risiko, Long-COVID-Merkmale zu entwickeln, bei Personen mit und ohne COVID-19-Impfung insgesamt sehr ähnlich war. Die Impfung verringerte nicht das Risiko für Angst/Depression, Kopfschmerzen, abdominale Symptome, Brust-/Halsschmerzen, Atembeschwerden und kognitive Symptome. Bestimmte Symptome, insbesondere Erschöpfung und Myalgien, traten jedoch bei der geimpften Bevölkerung seltener auf.

## Verbindung zu ME/CFS

Die erhöhte Inzidenz von Symptomen nach COVID-19 im Vergleich zur Influenza ist ein Hinweis auf eine gewisse Spezifität für die Art der Infektion. Es gibt jedoch auch einige Ähnlichkeiten zwischen Long COVID und myalgischer Enzephalomyelitis/chronischem Erschöpfungssyndrom (myalgic encephalomyelitis/chronic fatigue syndrome, ME/CFS). Wie Long COVID ist sie mit einer vorherigen Virusinfektion assoziiert, die häufig bei zuvor gesunden und aktiven Personen (hauptsächlich Frauen) auftritt [15]. Das chronische Krankheitsbild ist bei beiden Erkrankungen ähnlich, mit Erschöpfung, Benommenheit und Unwohlsein nach körperlicher Anstrengung (post-exertional malaise, PEM) [16], die die Aktivitäten des täglichen Lebens beeinträchtigen. Eine Studie hat gezeigt, dass von den Betroffenen, die nach 2 Monaten Long COVID symptomatisch blieben, 85% auch nach 1 Jahr noch über Symptome berichteten [17]. Long COVID kann nach 6 Monaten erhebliche Auswirkungen auf das Leben der Patienten haben, was in einigen Fällen zur Entwicklung einer ME/CFS-ähnlichen Erkrankung führen kann.

Eine lebensbeeinträchtigende Fatigue tritt in beiden Gruppen sowie bei Patienten mit chronischen Autoimmunerkrankungen [18] oder neurologischen Erkrankungen [19] sehr häufig auf. In einigen Fällen führt diese Erschöpfung zu schweren Beeinträchtigungen [20]. Patienten mit ME/CFS müssen ihr tägliches Aktivitätsniveau anpassen, um ein PEM zu vermeiden, das bei schwerem Verlauf zu Bettlägerigkeit mit Symptomen wie schwerer posturaler orthostatischer Intoleranz (severe postural orthostatic intolerance, POTS), Schlafstörungen, Myalgie, kognitiver Dysfunktion, Dysautonomie, neuroimmuno-endokriner Dysfunktion, Hyperakusis und Photophobie führen kann [21, 22].

Ähnlich wie bei Long COVID ist das Fehlen eines diagnostischen Tests eine große Hürde, sodass viele Patienten durch den Ausschluss anderer Erkrankungen diagnostiziert werden oder die Diagnose ME/CFS erst nach langer Zeit erhalten (>10 Jahre). Zwischen 80% und 90% der Patienten erhalten nie eine eindeutige Diagnose [23]. Die derzeitigen Behandlungsmöglichkeiten für ME/CFS sind begrenzt. Die abgestufte Bewegungstherapie wurde 2021 aus den Leitlinien des National Institute for Health and Care Excellence (NICE) gestrichen, da 50% der Patienten eine Verschlechterung ihres Zustands erlebt hatten [24, 25, 26]. Die Behandlung von Depressionen und anderen psychiatrischen Erkrankungen ist nur von begrenztem Nutzen.

Nach der Diagnose von ME/CFS erholen sich viele Patienten nie vollständig und werden von der Gesellschaft «vergessen». Die Belastung für die Familien ist jedoch enorm − viele Familien übernehmen eine lebenslange Pflegeverpflichtung. Ein besseres Verständnis von Long COVID, einer langwierigen Erkrankung mit vielen gleichen Symptomen wie bei ME/CFS, bietet daher die Chance, auch zum Verständnis und zur Behandlung von Patienten mit ME/CFS beitragen zu können, mit der Option zur Entwicklung therapeutischer Interventionen unter potenziell homogeneren Bedingungen.

Die ME/CFS-Forschung kann auch zum Verständnis der zugrunde liegenden pathophysiologischen Mechanismen beitragen, wie z.B. ein dysregulierter Energiestoffwechsel [27, 28, 29, 30], trainingsinduzierte Veränderungen des Plasmastoffwechsels [31], dysbiotische Darmbewegungen [32, 33] und die Dysfunktion von Immunzellen [34]. Bei Long-COVID-Patienten wurden auch Hinweise auf Stoffwechselstörungen und eine anhaltende Dysregulation des Immunsystems beobachtet [35]. Dies könnte möglicherweise auf eine virale Persistenz von SARS-CoV-2 oder anderen Viren zurückzuführen sein, obwohl dies bei ME/CFS-Patienten nicht nachgewiesen wurde [36]. Eine Persistenz von SARS-CoV-2 von 3–5 Monaten wurde bei immungeschwächten Patienten berichtet, jedoch ohne begleitende Symptome [37]; dies ist ein Bereich, der weiterer Untersuchungen bedarf [38].

Um Fortschritte zu erzielen, müssen Lehren aus der unzureichenden Behandlung von ME/CFS-Patienten gezogen und langfristige Forschungsprogramme aufgelegt werden, um die Biologie hinter Long COVID und ME/CFS vollständig zu verstehen. In Bevölkerungsgruppen, in denen der Krankheitsverlauf, das Fortschreiten der Krankheit und die Auswirkungen von Interventionen besonders intensiv untersucht wurden, können bestimmte Erkenntnisse möglicherweise früher gewonnen werden. Dazu gehören Personen im Spitzensport oder beim Militär, wo körperliche Fitness signifikant für den Erfolg ist und eine ressourcenintensive Leistungsüberwachung routinemäßig stattfindet.

## Long COVID in physisch aktiven Bevölkerungsgruppen

Mehrere epidemiologische Studien haben gezeigt, dass körperliche Inaktivität (d.h. weniger als nach den WHO-Empfehlungen für regelmäßige wöchentliche körperliche Aktivität) mit einem um 30% erhöhten Risiko einer Krankenhauseinweisung verbunden ist [39, 40], ähnlich wie ein schlecht eingestellter Diabetes. Vor dem Hintergrund dieser Erkenntnisse ist es nicht verwunderlich, dass sportlich aktive Menschen ohne chronische Erkrankungen in der Regel nur leichte akute Symptome entwickeln und nur selten eine stationäre Behandlung benötigen. Aber auch Berufssportler und internationale Leistungssportler können mit langwierigen Symptomen zu kämpfen haben, die sie oft an der Teilnahme an Wettkämpfen wie den Olympischen Spielen in Tokio hindern [41]. Bei einer anderen sehr aktiven Bevölkerungsgruppe, dem Militärpersonal, war ebenfalls bereits zu einem frühen Zeitpunkt der Pandemie klar, dass das Post-COVID-19-Krankheitsbild die Rolle und Einsatzfähigkeit der Streitkräfte Großbritanniens beeinträchtigen würde, auch wenn das Ausmaß nicht bekannt war. Das Ausmaß der Überwachung der körperlichen Gesundheit und die unterstützenden Trainings- und Rehabilitationsstrukturen für Leistungssportler und Militärpersonal bieten das Potenzial für einzigartige Einblicke in die Natur der Krankheit, die Pathophysiologie und in mögliche Behandlungsmethoden für Menschen, die an Long COVID leiden.

Die fragebogenbasierte AWARE-I-Studie an südafrikanischen Leistungssportlern zeigte, dass die akute und postakute COVID-Symptomatik bei aktiven Personen einem ähnlichen Muster folgt wie in der Allgemeinbevölkerung (*n* = 45) [42]. Die akute Erkrankung dauerte länger als bei anderen Ursachen von Atemwegsinfektionen, und eine Gruppe von 7 Symptomen («übermäßige Erschöpfung», «Schüttelfrost», «Fieber», «Kopfschmerzen», «veränderter/verminderter Geruchssinn», «Schmerzen/Druck in der Brust», «Atembeschwerden» und «Appetitlosigkeit») war mit einer längeren Wiederaufnahme der sportlichen Aktivität nach 40 Tagen verbunden. Ähnliche anhaltende Symptome wurde von Militärpersonal berichtet, darunter Erschöpfung, Husten, Atembeschwerden und Stimmungsschwankungen, die häufiger bei Personen über 40 Jahren auftraten [43, 44, 45].

Auch die Prävalenz persistierender Symptome ist ähnlich wie in der Allgemeinbevölkerung, wobei etwa 10% der Leistungssportler, die sich auf internationale Wettkämpfe vorbereiteten, >28 Tage (*n* = 147) unter Symptomen litten [10, 46], bei 27% die vollständige Rückkehr zum Sport nach 1 Monat verzögert war und 6% auch nach 90 Tagen noch beeinträchtigt waren. Diese Zahl war deutlich höher als die Daten der Studie vor COVID, in der nur 4% der Leistungssportler 1 Monat nach einer Atemwegserkrankung ihr Training noch nicht wiederaufgenommen hatten. Eine verzögerte Genesung trat doppelt so häufig bei Leistungssportlern mit Symptomen der unteren Atemwege auf (z.B. einschließlich Atembeschwerden ± Brustschmerzen). Neuere Daten amerikanischer College-Leistungssportler (*n* = 3597) zeigen eine deutlich geringere Prävalenz persistierender Symptome von nur 0,8% nach 28 Tagen [47]. Auch beim Militärpersonal nahm die Prävalenz der Long-COVID-Symptome mit der Zeit ab, wobei die Inzidenz zwischen der ersten Welle vor der Impfung (Wildtyp) und der zweiten Welle (Alpha-Variante) abnahm, was wahrscheinlich auf ein erhöhtes Krankheitsbewusstsein, eine bessere Verfügbarkeit von Selbstmanagementstrategien und die Dominanz der SARS-CoV-2-Variante zurückzuführen ist [44, 45, 48]. Weitere Studien sind in derselben Bevölkerungsgruppe der britischen Streitkräfte geplant, um die Auswirkungen der Immunisierung sowie der Delta- und Omicron-Varianten von SARS-CoV-2 zu verstehen. Die Impfung selbst wird von Leistungssportlern gut vertragen, und nur sehr wenige berichten über Auswirkungen der Impfung auf das Training [49].

Die Entwicklung von Interventionsstrategien hat möglicherweise dazu beigetragen, die Prävalenz lang anhaltender Symptome zu verringern. Wie bei anderen postviralen Erkrankungen beobachtet, bietet der vom NICE empfohlene Ansatz eines multimodalen und multidisziplinären Teams (MDT) einen umfassenden Nutzen für die Patienten bei der Behandlung lang anhaltender Symptome [50]. In den britischen Streitkräften wurden, nachdem der Bedarf zu einem frühen Zeitpunkt der Pandemie erkannt worden war, gleichzeitig mehrere verschiedene Wege beschritten, darunter eine Fernbeurteilung der Rehabilitation und anschließende Rehabilitationsprogramme, eine kombinierte Klinik für berufsbezogene und ärztliche Beurteilung und eine Längsschnittbeobachtungsstudie [44, 45, 51, 52, 53]. Die zentrale Frage lautete: Wie können wir einer körperlich aktiven Bevölkerung helfen, ihre volle Gesundheit und Aktivität wiederzuerlangen, und zwar auf breiter Basis und ohne Risiken?

Die Daten aus diesen Studien deuten darauf hin, dass etwa 2% der Soldaten nach COVID-19 Probleme entwickelten und zwei Drittel von ihnen eine MDT-Rehabilitation benötigten, die Aufklärung, pulmonale Rehabilitation, symptombezogene Aktivitätssteigerung, psychologische Beurteilung und Unterstützung sowie berufliche Unterstützung umfasste. Diese wurden in Form von Selbstmanagementprogrammen sowie einrichtungsbezogenen Programmen durchgeführt, gefolgt von einem fernbetreuten Selbstanleitungsprogramm. Drei Monate nach diesen Maßnahmen waren 91% der Teilnehmer wieder erwerbstätig, einige mit anhaltenden Symptomen, und fast alle wendeten die erlernten Strategien weiterhin an. Die Patienten erhielten eine Selbstmanagement-Broschüre mit Verweisen auf NHS-Ressourcen wie Your COVID Recovery. Im Verlauf der Pandemie wurden diese Maßnahmen zunehmend gemeindebasiert und von Hausärzten und anderen Angehörigen der Gesundheitsberufe unterstützt, wobei der Einsatz von Fachärzten auf schwere oder komplexe Fälle beschränkt blieb.

Kardiopulmonale Belastungstests (cardiopulmonary exercise testing, CPET) wurden als Eckpfeiler für das Verständnis des Ausmaßes und der Art der jeweiligen Einschränkung verwendet, da die meisten Post-COVID-19-Symptome mit Anstrengung verbunden sind oder sich durch Anstrengung verschlimmern [43]. Das CPET wurde im Rahmen einer ausführlichen klinischen Untersuchung eingesetzt, die auch Bluttests, EKG, Spirometrie, kognitive Beurteilung und einen 6-Minuten-Gehtest umfasste und bei Bedarf durch Computertomografie (CT)/Magnetresonanztomografie (MRT) ergänzt wurde [44, 45]. Erfreulicherweise wurden extrem niedrige Werte für kardiopulmonale Symptome festgestellt, was Bedenken hinsichtlich okkulter Organpathologien zerstreute [43] und mit den Daten von Leistungssportlern übereinstimmte. Ursprünglich wurde wegen der hohen Prävalenz von Myokarditis (d.h. etwa 25–50%) die Rückkehr zum Sport verzögert [54]. Diese Befürchtungen haben sich jedoch nicht bestätigt, da spätere Untersuchungen gezeigt haben, dass die Prävalenz klinischer Myokardereignisse bei Leistungssportlern ≤1% beträgt [55].

Studien in der Gruppe des Militärpersonals trugen ebenfalls dazu bei, potenzielle Faktoren zu identifizieren, die zu Long COVID beitragen. Dazu gehören objektive kognitive Beeinträchtigungen, die mit einem Altern um 10 Jahre oder mit der Überschreitung der britischen Promillegrenze korrespondieren, eine trainingsbedingte Dysautonomie bei bis zu 25% der Personen und eine trainingsinduzierte ventilatorische Ineffizienz [43, 56]. Ähnliche Veränderungen wurden auch bei Leistungssportlern nach COVID-19 beobachtet, wobei sich die Veränderungen sowohl der kardiovaskulären als auch der respiratorischen Reaktionsparameter auf die kardiorespiratorische Leistung während der CPET auswirkten [57]. Leistungssportler mit Long COVID zeigen ebenfalls Symptome von Dysautonomie und ventilatorischer Ineffizienz. Sie berichten über Störungen sowohl der Ruheherzfrequenz (Ruhe-HF) als auch der Herzreaktion bei submaximaler Belastung sowie über eine «unbefriedigende Atmung» und ein scheinbar unverhältnismäßig hohes Maß an Atembeschwerden. Einige dieser klinischen Merkmale ähneln dem Zustand des unerklärlichen Leistungsabfalls oder des Übertrainingssyndroms, z.B. Erschöpfung und mangelnde Erholung [58]. Die genauen pathobiologischen Faktoren, die diesem Syndrom zugrunde liegen, sind noch schwer zu bestimmen, aber die Ähnlichkeiten dieser Pathophysiologie mit Long COVID rechtfertigen weitere Untersuchungen.

Um die mittelfristigen Auswirkungen von COVID-19 auf die britischen Streitkräfte zu verstehen, wurde die longitudinale Kohortenstudie «Military COVID-19, Observational outcomes in a Viral Infectious Disease (M-COVID)» (1061/MODREC/20) initiiert. Erste Ergebnisse zeigen, dass sich vollständig genesene, in der Gemeinschaft lebende Personen 5 Monate nach der Erkrankung in keinem Parameter von einer Kontrollpopulation unterscheiden, dies gilt jedoch nicht für Personen, die sich von einer Krankenhauserkrankung erholt haben [59, 60]. Diese Ergebnisse legen nahe, dass sich Personen mit anfänglich schweren oder persistierenden Symptomen sicherheitshalber geeigneten Untersuchungen wie dem CPET unterziehen sollten, bevor sie wieder anstrengende körperliche Aktivitäten aufnehmen.

Insgesamt deuten die Ergebnisse in dieser Bevölkerungsgruppe körperlich aktiver Menschen darauf hin, dass der Verlauf und der Anteil von Long COVID ähnlich sind wie in anderen Bevölkerungsgruppen, obwohl die anfängliche Erkrankung insgesamt viel weniger schwerwiegend ist. Personen, die eine leichte bis mittelschwere Erkrankung hatten und sich scheinbar vollständig erholt haben, sind in der Tat mit hoher Wahrscheinlichkeit genesen, sodass Ressourcen für Personen mit einer längeren oder anfänglich sehr schweren Erkrankung freigesetzt werden können. Eine ambulante oder häusliche Rehabilitation kann für die meisten Menschen von Nutzen sein, wobei eine detaillierte Beurteilung, einschließlich CPET, hilfreich ist, um spezifische Einschränkungen zu erkennen.

## Was sagt uns der kardiopulmonale Belastungstest?

Wie bereits erwähnt, ist der CPET ein wertvolles Instrument, um die Leistungseinschränkungen von Einzelpersonen und somit die Art der erforderlichen Rehabilitation zu verstehen. Er ist aber auch als Forschungsinstrument nützlich, um die verschiedenen physiologischen Prozesse zu verstehen, die bei Long COVID dysfunktional werden.

Eine isolierte Betrachtung von Long COVID durch die medizinischen Einzeldisziplinen wird wahrscheinlich nicht zu einem einheitlichen Verständnis der zugrunde liegenden pathologischen Mechanismen führen. Wie bereits erwähnt, ist sowohl die Belastungseinschränkung als auch die Erschöpfung von großer Bedeutung für die beschriebenen Symptome [61], und ihre Untersuchung erfordert einen integrierten physiologischen Ansatz bezogen auf den gesamten Körper. Der CPET ist eine robuste, validierte und erprobte Methode zur Bewertung der Reaktion gleich mehrerer Systeme auf körperliche Aktivität und wurde als Mittel zur Entschlüsselung der Mechanismen erkannt, die Long COVID zugrunde liegen. Die veröffentlichte Evidenz ist jedoch dadurch eingeschränkt, dass die meisten Studien Patientenkohorten nach einem Krankenhausaufenthalt betreffen. Diese Kohorten unterscheiden sich signifikant von einer nicht hospitalisierten Long-COVID-Gruppe [61], und eine Einschränkung der körperlichen Leistungsfähigkeit im Rahmen einer schwereren akuten Infektion ist unabhängig von der COVID-19-spezifischen Pathologie zu erwarten [62, 63]. Es könnte jedoch argumentiert werden, dass eine SARS-CoV-2-Symptomatik, die signifikant genug ist, um lang anhaltende Symptome in einer Bevölkerungsgruppe mit vormals «leichter Erkrankung» zu verursachen, auch bei Personen mit einer schwereren Ersterkrankung auftreten sollte. Daher könnten Studien an Letzteren nützliche Informationen liefern.

Rinaldo und Kollegen untersuchten eine Gruppe von Patienten 3 Monate nach der Entlassung aus dem Krankenhaus [64]. Es gab keine offensichtlichen Atmungseinschränkungen, obwohl 55% einen reduzierten maximalen Sauerstoffverbrauch (*V̇*_O2_) in einem überzeugenden maximalen Testprotokoll aufwiesen. Die Verfasser kamen zu dem Schluss, dass das Muster der frühen anaeroben Schwelle, des verringerten Sauerstoffpulses und des reduzierten *V̇*_O2_/Arbeitsleistung-Verhältnisses auf Dekonditionierung zurückzuführen sei. Dekonditionierung ist jedoch eine ungenaue Diagnose − in der gängigen Praxis wird damit ein passiver Prozess der muskulären Ineffizienz bezeichnet, der häufig auf eine verminderte Nutzung (z.B. Immobilität, Krankheit) zurückzuführen ist. Ohne eine direkte Messung des Herzzeitvolumens oder der peripheren Sauerstoffaufnahme kann die Möglichkeit einer verminderten Sauerstoffversorgung oder einer aktiveren Ätiologie der peripheren Muskelpathologie jedoch nicht ausgeschlossen werden. Bei der Entlassung aus dem Krankenhaus wurde eine periphere Muskelfunktionsstörung [65] mittels Belastungsechokardiogramm, arterieller Blutentnahme und Lösung der Fick-Formel zur Messung des Herzzeitvolumens und der peripheren Sauerstoffextraktion nachgewiesen. Der *V̇*_O2_-Spitzenwerte korrelierte mit dem Schweregrad der Erkrankung und eine Abnahme des *V̇*_O2_/-Spitzenwerts korrelierte mit einer Abnahme der peripheren Sauerstoffaufnahme, wobei sich die Pathologie auf die peripheren Bewegungsmuskeln beschränkte. In dieser Studie wurde auch eine verminderte Atemeffizienz festgestellt, eine Kombination aus erhöhter Atemfrequenz und Tot­raumventilation, sowie eine erhöhte Chemosensitivität, ein Ergebnis, das auch in anderen Studien nach 3 Monaten beobachtet wurde [66, 143].

Die Einschränkung der körperlichen Leistungsfähigkeit bleibt in der Kohorte nach dem Krankenhausaufenthalt bestehen, wobei etwa 20% der Personen nach 6 Monaten noch einen *V̇*_O2_-Spitzenwert von weniger als 85% des vorhergesagten Maximums aufweisen [66]. Immer mehr Hinweise bestätigen eine ähnliche Einschränkung für hausärztlich betreute Long-COVID-Fälle [56, 67, 68]. Obwohl die Studie von Singh und Kollegen [68] an noch symptomatischen Personen, die nach 12 Monaten hausärztlich behandelt wurden, klein ist (*n* = 10, mit angepassten Kontrollen), trägt sie wesentlich zum Verständnis bei. Mithilfe eines invasiven CPET-Protokolls maßen die Forscher direkt das Herzzeitvolumen, die Sauerstoffversorgung und die Sauerstoffaufnahme. Bei Patienten mit einem *V̇*_O2_-Spitzenwert über 75% war die körperliche Leistungsfähigkeit eingeschränkt, da die Sauerstoffaufnahme in der Peripherie nicht gesteigert werden konnte. De Boer und Kollegen vermuten, dass dies auf eine mitochondriale Dysfunktion zurückzuführen ist, die sich aus einer offensichtlichen Verringerung der Fettsäureoxidation während des Trainings ableiten lässt [67]. Die Kontrolldaten in dieser Studie sind jedoch historisch und schlecht abgestimmt, sodass an dieser Stelle keine eindeutigen Schlussfolgerungen gezogen werden können. Angesichts der Hinweise auf direkte und indirekte Mechanismen der mitochondrialen Schädigung bei COVID-19 [69, 70, 71] erscheinen jedoch weitere Untersuchungen dieser Hypothese angebracht.

Schließlich wurde in diesen Studien, wie auch in anderen oben erwähnten Bevölkerungsgruppen, eine autonome Dysfunktion festgestellt. Bei Patienten mit Long COVID wurde eine Abnahme der Spitzen-HF [72], der chronotropen Inkompetenz [73] und der Dysautonomie [56] dokumentiert, jedoch nicht immer in Verbindung mit dem gleichen Muster der CPET-Einschränkung der peripheren Muskulatur wie oben beschrieben, was darauf hindeutet, dass die Dysautonomie ein zusätzlicher einschränkender Mechanismus sein könnte.

Diese Daten aus CPET-Studien weisen auf einen Mechanismus der peripheren Muskulatur hin, der bei einer beträchtlichen Anzahl von Personen zu Bewegungsstörungen führt. Ein besseres Verständnis dieses Mechanismus durch weitere Phänotypisierung könnte das Verständnis anderer Symptome dieser schwächenden Krankheit verbessern. Es scheint jedoch, dass mehrere Prozesse beteiligt sind, einschließlich einer ineffizienten Atmung und einer autonomen Dysfunktion. Die Identifizierung des Vorhandenseins und des Zusammenspiels dieser Mechanismen bei einer Person dürfte sowohl für das Verständnis ihrer Symptomzusammensetzung als auch für die Personalisierung einer gezielten Therapie wichtig sein.

## Dysautonomie POTS

Wie bereits erwähnt, scheint die Dysautonomie in verschiedenen Bevölkerungsgruppen ein Merkmal von Long COVID zu sein, das häufig mit Veränderungen der HF-Regulation einhergeht. Auf Synkopen und Dysautonomien spezialisierte Kliniken auf der ganzen Welt, darunter auch die von zwei der Autoren (M.D., P.B.L.), berichten über Fälle von kardiovaskulären autonomen Störungen und peripherer autonomer Neuropathie während und nach einer COVID-Infektion [74, 75, 76, 77, 78]. Typische Symptome nach COVID sind Atembeschwerden, Palpitationen und Schwindel, der sich im Stehen deutlich verschlechtert (Orthostase). Dazu gehört das posturale orthostatische Tachykardiesyndrom (POTS), ein Zustand, der durch einen Anstieg der Herzfrequenz auf >30 Schläge pro Minute (bpm) im Stehen ohne Blutdruckabfall gekennzeichnet ist und von den oben genannten Symptomen begleitet wird [79].

Wir untersuchten systematisch die Kipptischtestreaktionen von 27 überwiesenen Patienten mit lang andauernden autonomen Cochlea-Insuffizienzen. Es wurden unterschiedliche Muster hämodynamischer Reaktionen auf das Stehen beobachtet:

1. POTS − 15% der Kohorte
2. POTS, niedriger oder normaler Blutdruck im Ausgangszustand − 4% der Kohorte
3. POTS unterhalb der Schwelle (anhaltende Erhöhung der Herzfrequenz <30 bpm, aber mehr als 15% Erhöhung gegenüber dem Ausgangswert), mit Hypertonie im Ausgangszustand: 33% der Kohorte
4. POTS unter dem Schwellenwert, niedriger oder normaler Ausgangsblutdruck: 30% der Kohorte
5. Niedriger Ausgangsblutdruck (systolischer Blutdruck <100 mmHg in Rückenlage), kein signifikanter Anstieg der HF oder des Blutdrucks − 4% der Kohorte
6. Innerhalb der normalen Grenzen: 15% der Kohorte

Es gab Anzeichen für eine erhöhte Aktivität des sympathischen Nervensystems: Bei 85% der Patienten stieg die Herzfrequenz im Stehen um mehr als 15% und bei 30% der Patienten lag der Blutdruck über 130/80 mmHg. Die meisten (78%) zeigten Blutdruckschwankungen im Stehen (Blutdruckschwankungen mit einem systolischen Spitzenwert von >30 mmHg über 120 s), ein Prädiktor für eine drohende vasovagale Synkope [80], was auf eine hämodynamische Instabilität hinweist [81, 82].

Diese Ergebnisse bestätigen die Ergebnisse anderer Gruppen, die von unterschiedlichen POTS-Raten zwischen 22% und 75% berichten [75, 78]. Im Gegensatz zu anderen Gruppen [78, 83] hatte jedoch kein Patient dieser Kohorte eine orthostatische Hypotonie, obwohl 22% einen systolischen Blutdruck von <100 mmHg während der Rückenlagephase aufwiesen. Dies könnte auf ein Bias aufgrund der Überweisung an die kardiologische Abteilung oder auf die geringe Teilnehmerzahl an dieser Studie zurückzuführen sein.

Obwohl es sich bei diesen Ergebnissen um eine kleine Gruppe von Patienten handelt, die speziell für einen Kipptischtest überwiesen wurden, ist die Überaktivität des Sympathikus eindeutig erwiesen, da bei den meisten Betroffenen die Herzfrequenz und der Blutdruck im Stehen ansteigen. Dies deutet auf eine autonome Dysregulation hin, die entweder nach der Infektion neu aufgetreten ist oder durch die Infektion demaskiert wurde.

Zu den Medikamenten, die üblicherweise bei autonomen Herz-Kreislauf-Störungen wie POTS eingesetzt werden, gehören Flüssigkeitsexpander (Fludrocortison) und α-Agonisten (Midodrin), die den Blutdruck erhöhen, den venösen Rückfluss zum Herzen verbessern und dadurch die übermäßige sympathische Reaktion reduzieren. Darüber hinaus können β-Rezeptorenblocker die Symptome einer übermäßigen sympathischen und adrenergen Reaktion auf die Orthostase lindern. Die Ergebnisse dieser kleinen Kohorte deuten darauf hin, dass die erstgenannten Medikamente für diese Untergruppe möglicherweise nicht indiziert sind und β-Blocker hilfreicher sein könnten. Darüber hinaus profitieren diese Patienten wahrscheinlich von Maßnahmen zur Modulation des autonomen Nervensystems, die speziell die Sympathikusaktivität reduzieren und den Vagustonus erhöhen, wie z.B. Atemtraining, Biofeedbacktraining der Herzfrequenzvariabilität (HFV) und rhythmische Körperhaltungsübungen, die die Atmung einbeziehen, wie z.B. Yoga.

## Neurologische Beiträge zu Long COVID

Das Auftreten von Dysautonomie und Hirnnebel deutet auf neurologische Beiträge zu Long COVID hin, und Erschöpfung/Fatigue, eines der häufigsten Symptome nach einer COVID-Erkrankung, könnte ebenfalls durch eine neurologische Dysfunktion beeinflusst werden. Um dies zu untersuchen, wurde die Dysfunktion neuronaler Schaltkreise bei Patienten untersucht, die mindestens 6 Wochen nach einer leichten oder mittelschweren COVID-19-Infektion unter Erschöpfung litten.

Entzündungen sind wahrscheinlich entscheidend für die Entstehung von COVID-19-Folgeschäden. Bei Personen mit langer COVID-Dauer sind die Entzündungsmarker über mehrere Monate erhöht [35]. Es gibt mehrere Wege, auf denen das Immunsystem das Nervensystem physiologisch beeinflussen kann und umgekehrt [84]. Unabhängig vom molekularen Auslöser müssen auf jeden Fall neuromuskuläre Mechanismen zur symptomatischen Erschöpfung beitragen, da die häufigsten Symptome der Post-COVID-Erschöpfung mit körperlicher und kognitiver Aktivität zusammenhängen, die beide auf neuronalen Schaltkreisen beruhen; welche neuronalen Systeme betroffen sind, ist jedoch nicht bekannt.

Freiwillige Teilnehmer mit selbstberichteter Post-COVID-Erschöpfung wurden einer Reihe von Verhaltens- und neurophysiologischen Tests unterzogen, bei denen das zentrale, das periphere und das autonome Nervensystem untersucht wurden [85]. Im Vergleich zu alters- und geschlechtsgleichen Studienteilnehmern ohne Erschöpfung zeigten sich Unterschiede in bestimmten neuronalen Schaltkreisen: Der primäre motorische Kortex (M1), einer der wichtigsten Bereiche für willkürliche Bewegungen und die Ansteuerung von Muskeln, war bei der Kohorte mit Erschöpfung weniger erregbar. Studienteilnehmer mit Erschöpfung hatten auch eine höhere Herzfrequenz und eine niedrigere HFV, beides Phänomene, die mit Dysautonomie in Verbindung gebracht werden, die häufig mit Erschöpfung einhergeht. Sensorische Rückkopplungskreise und Störungen absteigender neuromodulatorischer Kontrollsysteme waren nicht betroffen. Schließlich wurden myopathische Veränderungen in der Skelettmuskulatur beobachtet: Obwohl die Kohorte mit Erschöpfung ein normales Kraftniveau aufwies, war die Fähigkeit des Muskels, nach anhaltender Kontraktion Kraft zu erzeugen, im Vergleich zu den Kontrollteilnehmern reduziert.

Diese Anomalien in objektiven Tests können neue Wege für gezielte therapeutische Interventionen aufzeigen und könnten als schnelle und zuverlässige Biomarker für die Diagnose und Überwachung des Fortschreitens der Erschöpfung im Laufe der Zeit dienen.

Die Dysautonomie äußert sich in einer erhöhten Aktivität des Sympathikus gegenüber dem Parasympathikus und wird mit der Erschöpfung bei anderen Autoimmunerkrankungen in Verbindung gebracht. Die zunehmenden Hinweise auf eine vagale Dysregulation nach COVID-19 [86, 87] legen nahe, dass eine zu geringe Aktivierung des Nervus vagus die Ursache dieser Dysautonomie sein könnte. Neuere Studien haben gezeigt, dass eine tägliche nicht invasive Vagusnervstimulation (nVNS) über 4–5 Wochen die Erschöpfung und Entzündungsmarker bei Patienten mit autoimmuner Erschöpfung reduziert [88, 89]. Dies unterstützt die Hypothese, dass die vagale Hypoaktivität eine Ursache für die Erschöpfung und die nVNS eine wirksame Therapie gegen Fatigue sein könnte. Ein ähnlicher Ansatz wird derzeit bei Patienten mit Post-COVID-Fatigue untersucht. Dabei werden die Auswirkungen der Stimulation des Vagusnervs über den aurikulären Zweig auf die Erschöpfung sowie die physiologischen, neurophysiologischen, verhaltensbezogenen und immunologischen Korrelate der Fatigue untersucht.

Obwohl SARS-CoV-2 in erster Linie eine Atemwegsinfektion verursacht, handelt es sich bei COVID um eine Multisystemerkrankung [90], die auch das Nervensystem befällt. Das Verständnis der Auswirkungen der Dysfunktion auf die verschiedenen interagierenden Organsysteme ist wichtig für das Verständnis dieser Krankheit. Die Entdeckung abnormaler subzellulärer Funktionsänderungen ist wahrscheinlich entscheidend für die Entwicklung wirksamer Behandlungsstrategien.

## Mikrogerinnsel und endotheliale Dysfunktion

Mögliche mechanistische Erklärungen für die Erschöpfung und andere Long-COVID-Symptome sind Anomalien in der Sauerstoffverfügbarkeit des Gewebes aufgrund vaskulärer Dysfunktion und Hyperkoagulation sowie eine mitochondriale Dysfunktion, die die zelluläre Bioenergetik stört. Gerinnungsstörungen und endotheliale Dysfunktion sind die wichtigsten pathologischen Befunde bei akuter und postakuter COVID-19 [91, 92, 93, 94], wobei das Spike-Protein von SARS-CoV-2 möglicherweise durch Gerinnungsfaktoren aktiviert wird [95]. Diese pathologischen Befunde sind sogar bei geimpften Personen erhöht [96] und sind wahrscheinlich die Ursache für das erhöhte Risiko kardiovaskulärer Ereignisse bei COVID-19-Genesenen [97]. Daher ist es wichtig zu verstehen, wie die endotheliale Dysfunktion und Koagulopathien entstehen und wie sie sich auf die Gewebefunktion auswirken.

Es gibt auch immer mehr Hinweise darauf, dass virale Produkte, Immunzellen und/oder Entzündungsmediatoren eine zentrale Rolle bei den pathophysiologischen Mechanismen spielen, die die persistierenden Symptome von Long COVID verursachen. Mehrere Studien haben gezeigt, dass SARS-CoV-2-RNA mehrere Monate nach einer akuten Infektion persistiert, obwohl dies noch nicht eindeutig mit einer Long-COVID-Symptomatik in Verbindung gebracht wurde. Eine akute SARS-CoV-2-Infektion kann das Immunsystem dysregulieren und die Reaktivierung anderer persistierender Viren ermöglichen [98].

Thrombozyten und Endothelzellen können mit viralen Produkten und zirkulierenden Entzündungsmolekülen interagieren und dadurch eine Hyperkoagulation auslösen [99, 100, 101], die kleine Blutgefäße blockiert und die Sauerstoffversorgung beeinträchtigt. Entzündungssignale, die von einem dysfunktionellen Endothel ausgehen, können Gerinnungskaskaden auslösen [102]. Antikörper von Patienten mit einer schweren COVID-19-Erkrankung können auch prokoagulierende Thrombozyten und die Apoptose von Thrombozyten induzieren [103]. Alternativ kann die Hyperkoagulation auch durch SARS-CoV-2 selbst ausgelöst werden: Das Spike-Protein S1 von SARS-CoV-2 aktiviert Thrombozyten und verstärkt so Entzündungssignale, einschließlich einer erhöhten Zytokinproduktion durch Monozyten [104, 105]. S1 induziert auch strukturelle Veränderungen in blutgebundenen Molekülen, einschließlich des löslichen Plasmaproteins Fibrinogen, wodurch deren Aggregation verstärkt wird und sie resistent gegen Trypsinisierung werden [106]. Eine solche Aggregation kann auch durch Protein-Protein-Interaktionen zwischen Fibrinogen und anderen Viren oder Entzündungsmolekülen ausgelöst werden [107].

**Abb. 1:**
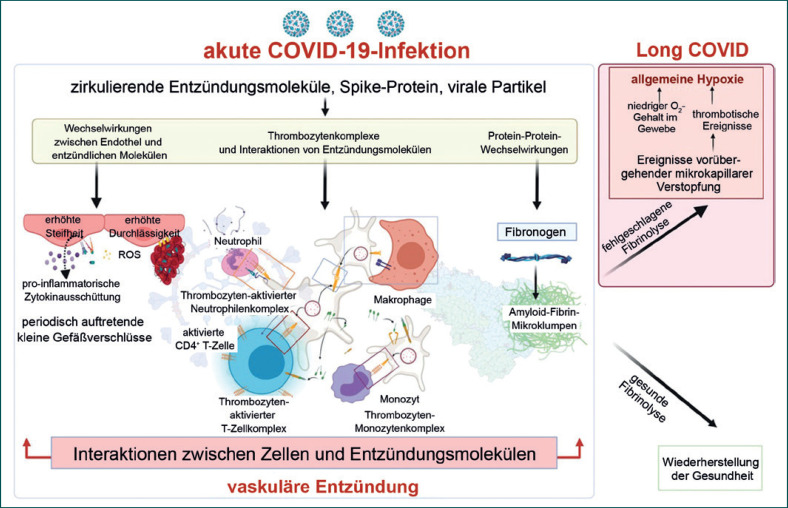
Pathologische Befunde zur Gerinnung bei Long COVID. ROS = reaktive Sauerstoffspezies. Erstellt mit BioRender.com.

Mikrogerinnsel, ähnlich denen, die durch S1 ausgelöst werden, wurden kürzlich im Blutkreislauf von Patienten mit Long COVID gefunden [92]. Diese Mikrogerinnsel sind resistent gegen Fibrinolyse und enthalten zahlreiche Entzündungsmoleküle, die sowohl die Gerinnungspathologie als auch die systemische Endothelitis aufrechterhalten können. Zu den in diesen Gerinnseln eingeschlossenen Molekülen gehören Fibrinogen, von-Willebrand-Faktor, α2-Antiplasmin (verhindert den Abbau von Blutgerinnseln durch die typischen fibrinolytischen Prozesse) und Plasminogen-Aktivator-Inhibitor Typ 1 [92]. Die Folge ist eine gestörte Gerinnungsphysiologie, die letztlich zu einer systemischen Gewebeischämie und Hypoxie führen kann (Abb 1).

Ein weit verbreiteter zellulärer Sauerstoffmangel kann zu vielen der persistierenden Symptome führen, die bei Long COVID beobachtet werden, und könnte die oben erwähnte Verringerung des Sauerstoffverbrauchs nach dem CPET erklären. Die weitere Erforschung dieser Mechanismen und potenzieller Maßnahmen zu ihrer Bekämpfung wird für die Behandlung von Long COVID von Bedeutung sein. Dies wurde auch im jüngsten Bericht des US Government Accountability Office [108] anerkannt, in dem Autoimmunreaktionen, Viruspersistenz, Organschäden und Mi­kroaggregation als wichtige Forschungsbereiche im Zusammenhang mit Long COVID genannt werden.

## Mitochondriale Dysfunktion und Erschöpfung

Neben einer gestörten Sauerstoffversorgung des Gewebes kann der in die Zelle gelangte Sauerstoff aufgrund einer gestörten Mitochondrienfunktion möglicherweise nicht ausreichend ATP bilden. Frühere Studien haben aufgrund experimenteller Beobachtungen die Hypothese aufgestellt, dass SARS-CoV-2 in der Lage ist, Mitochondrien zu kapern und für sein Überleben zu nutzen [16]. Die Aktivierung des Inflammasoms durch eine Schädigung der Mitochondrien führt zu einer ineffektiven Interferonproduktion als Reaktion auf eine Virusinfektion, zu erhöhtem oxidativen Stress und in einigen Fällen zu einer verlängerten Aktivierung des körpereigenen Immunsystems [16, 109]. Da sich das Virus in den Mitochondrien repliziert, kann sich deren Stoffwechselleistung verändern, was zu einer verstärkten Entzündungsreaktion führt, die die Schwere der Erkrankung verschlimmert. Experimentelle Studien von Ajaz et al. [110] und anderen [111] bestätigten Hinweise auf eine mitochondriale Dysfunktion, metabolische Veränderungen mit erhöhter Glykolyse und erhöhte Konzentrationen von Mitokinen in mononukleären Zellen des peripheren Blutes von COVID-19-Patienten, wobei Letztere mit dem Schweregrad der Erkrankung korrelierten. Eine wirtsabhängige Dysregulation der Glykolyse und des mitochondrialen Stoffwechsels wurde auch durch die Analyse von Transkriptomdaten aus Nasopharyngealabstrichen, peripheren mononukleären Blutzellen, Lungenbiopsien und bronchoalveolärer Lavageflüssigkeit von COVID-19-Patienten nachgewiesen [112].

Die CPET-Ergebnisse unterstützen auch einen dysfunktionalen Stoffwechsel, der zur Bewegungseinschränkung nach COVID-19 beiträgt. In mehreren Post-COVID-CPET-Studien wurde unabhängig von kardiopulmonalen Einschränkungen über einen reduzierten Spitzenwert des Sauerstoffverbrauchs berichtet [65, 66, 68, 72, 113], was auf einen verminderten Spitzenwert der Sauerstoffaufnahme [68] und möglicherweise auf eine Beeinträchtigung der Fettsäureoxidation [67] hinweist. Während eine verminderte Sauerstoffversorgung aufgrund einer veränderten peripheren vasomotorischen Reaktion [68, 114] eine mögliche Erklärung für diese Befunde liefern kann (sekundär zu einer autonomen und endothelialen Dysfunktion), bleibt eine veränderte mitochondriale Funktion ebenfalls eine Möglichkeit.

Die metabolische Bildgebung mit nicht invasiven Techniken wie der ^31^P-Magnetresonanzspektroskopie (^31^P-MRS) des Skelettmuskels und des Herzens hat Anomalien der mitochondrialen Atmung bei eng verwandten Erkrankungen wie ME/CFS gezeigt [115, 116, 117]. Bei Patienten mit Fatigue-dominierter Long COVID wurde eine beeinträchtigte oxidative Phosphorylierung und ein erhöhter pH-Wert der Skelettmuskulatur in der ^31^P-MRS nachgewiesen (B. Raman et al., unveröffentlichte Arbeit), was die Möglichkeit aufwirft, dass eine ähnliche mitochondriale Dysfunktion auch zu Long COVID beiträgt.

Bei ME/CFS-Patienten scheinen viele Aspekte der mitochondrialen Funktion und des Stoffwechsels verändert zu sein. Es gibt Berichte über eine abnorme Aktivität der mitochondrialen Atmungskette und der Pyruvat-Dehydrogenase [118, 119], eine Ineffizienz von Komplex V [27, 28], ein beeinträchtigtes ATP-Profil der Neutrophilen [120] sowie über einen veränderten Sauerstoffverbrauch lebender ausplattierter Zellen [29, 121]. Diese Daten in Verbindung mit Erkenntnissen aus umfangreichen Proteom-, Transkriptom- [122] und Metabolomstudien [119, 123, 124, 125] sprechen für eine energetische Ursache der Symptome bei ME/CFS-Patienten. Zukünftige Forschung muss klären, ob ähnliche Veränderungen bei Patienten mit Long COVID auftreten und ob metabolische Therapien, die die mitochondriale Funktion wiederherstellen, für die Patienten von Nutzen sein könnten.

Verschiedene (pharmakologische und nicht pharmakologische) Therapien haben ihr Potenzial zur Verbesserung der mitochon­drialen Funktion in vergleichbaren Krankheitsmodellen gezeigt. Dazu gehören Coenzym Q10 [126], α-Liponsäure und Acetyl-L-Carnitin [127], NADH [128, 129, 130], Resveratrol [131], Methylphenidat-Hydrochlorid [132], *N*-Acetylcystein [127, 133], Ubichinol, Vitamin E [127], sorgfältig maßgeschneiderte Rehabilitationsprogramme und andere. Derzeit laufen zahlreiche Studien, in denen die Wirksamkeit einiger dieser Medikamente bei Patienten mit Long COVID untersucht wird (siehe https://clinicaltrials.gov/ct2/home). Neu hinzugekommen sind endogene Stoffwechselmodulatoren, eine Mischung aus verzweigtkettigen Aminosäuren und Aminosäurederivaten, die in unterschiedlichen Verhältnissen generiert wurden, um mehrere biologische Stoffwechselwege in den Ausganszustand zurückzusetzen, die zelluläre Energieversorgung zu verbessern und die Homöostase wiederherzustellen, um die mitochondriale Funktion zu optimieren. Ein Beispiel hierfür ist AXA-1125, das nachweislich Stoffwechsel- und Entzündungsprozesse bei Patienten mit nicht alkoholischer Steatohepatitis verbessert [134]. Derzeit läuft in Großbritannien eine randomisierte, doppelblinde, placebokontrollierte klinische Studie zur Bewertung der Wirksamkeit dieser Therapie bei Patienten mit Fatigue-dominierter Long COVID. Im Rahmen der Studie soll die Hypothese getestet werden, ob die Wiederherstellung des mitochondrialen Stoffwechsels den Erschöpfungszustand bei Patienten mit Long COVID verbessert.

Long COVID ist eine Multisystemerkrankung, die zu Funktionsstörungen des Atem-, Herz- und Nervengewebes führt, die wohl zumindest teilweise auf Veränderungen des zellulären Energiestoffwechsels und eine verminderte Sauerstoffversorgung des Gewebes zurückzuführen sind. Erfolgreiche Behandlungsstrategien werden daher wahrscheinlich direkt oder indirekt auf diese Veränderungen abzielen.

## Fernbetreutes inspiratorisches Muskeltraining zur Behandlung von Long COVID

Viele Menschen, die sich von COVID-19 erholen, leiden unter lang anhaltenden Symptomen, einschließlich Atembeschwerden, die die Aktivitäten des täglichen Lebens einschränken. Die Rückkehr zur «Normalität», sei es bei den Aktivitäten des täglichen Lebens, bei der Arbeit oder beim Sport, ist häufig mit einer Verschlimmerung der Symptome verbunden, was für Personen, die sich von COVID-19 erholen, eine Reihe von Herausforderungen mit sich bringt [135]. Angesichts der großen Zahl der Betroffenen müssen sichere, effektive und nachhaltige Rehabilitationsstrategien entwickelt werden.

Inspiratorisches Muskeltraining (IMT) nutzt die Atmung mit reduziertem Luftstrom, um in der Atemmuskulatur eine hypertrophe Reaktion hervorzurufen, die der in der peripheren Muskulatur nach einem Krafttrainingsprogramm ähnelt [136]. IMT führt nachweislich zu klinisch bedeutsamen Verbesserungen der Atemnot und der Lebensqualität bei Patienten mit chronisch obstruktiver Lungenerkrankung (chronic-obstructive pulmonary disease, COPD) [137] und wird von Patienten mit Bronchiektasen gut vertragen und als vorteilhaft empfunden [138]. Da unzureichende Behandlungsergebnisse nach einer COVID-19-Infektion zum Teil durch eine Schwäche der Atemmuskulatur bedingt sind [139], könnte das IMT eine praktikable häusliche Rehabilitationsmethode darstellen.

Ziel des IMT-Projekts war es, das Potenzial von häuslichem IMT zur Verbesserung und Beschleunigung der Genesung von COVID-19 zu evaluieren. Konkret untersuchte die Studie den Einfluss von 8 Wochen IMT auf die Atemfunktion, die Atembeschwerden, die körperliche Belastungstoleranz, die tägliche körperliche Aktivität und die Wahrnehmung von Gesundheit und Wohlbefinden [140]. Teilnehmer, die mindestens 4 Wochen nach der COVID-19-Infektion an persistierenden Symptomen litten, wurden nach dem Zufallsprinzip mit einer Gewichtung von 4:1 entweder der Interventions- oder der Kontrollgruppe zugeteilt. IMT war mit einer signifikanten und klinisch bedeutsamen Verbesserung der gesundheitsbezogenen Lebensqualität in allen Teilbereichen einschließlich Atembeschwerden assoziiert (IMT: *n* = 111, Kontrollen: *n* = 37). Das Ausmaß der Verbesserung des Schweregrads der Atembeschwerden war doppelt so hoch wie der als klinisch relevant angesehene Wert. IMT verbesserte auch die Kraft der Atemmuskulatur, die geschätzte aerobe Fitness und das Ausmaß moderater körperlicher Aktivität bei gleichzeitiger Verringerung der im Sitzen verbrachten Zeit. Daher könnte IMT eine wichtige COVID-19-Rehabilitationsstrategie für die häusliche Behandlung darstellen. Weitere Untersuchungen zu den unterschiedlichen Rehabilitationsreaktionen sind aufgrund der Diversität von Long Covid notwendig. Die in dieser Studie beobachtete signifikante Abbruchrate (37% der Teilnehmer an der Intervention nahmen nicht an der Post-IMT-Sitzung teil) zeigt, dass es wahrscheinlich keine Strategie gibt, die für alle geeignet ist. Um dieser Variabilität zu begegnen, sind größere Studien besonders wertvoll, in denen verschiedene Muster von Symptomen und Reaktionen auf mehrere verschiedene Interventionen verfolgt werden, z.B. im Rahmen integrierter Versorgungspfade.

## STIMULATE-ICP

Integrierte Versorgungspfade (Integrated Care Pathways, ICP) haben sich bei der Behandlung mehrerer langwieriger Erkrankungen als wirksam erwiesen, da sie den gesamten Versorgungspfad von der Primärversorgung über das MDT bis hin zur Sekundärversorgung abdecken [141]. Mit dem ICP-Ansatz wäre es möglich, Long COVID ganzheitlich zu untersuchen und zu behandeln, was die dringend benötigte Realisierung skalierbarer und verallgemeinerbarer Lösungen erleichtern würde.

Das vom NIHR finanzierte Programm STIMULATE-ICP (Symptoms, Trajectory, Inequalities and Management: Understanding Long-COVID to Address and Transform Existing Integrated Care Pathways) wird Kliniker und Wissenschaftler mit Wissen versorgen, politischen Entscheidungsträgern Fakten liefern und die Patientenversorgung verbessern, während gleichzeitig reale Daten in großem Umfang gesammelt werden [142]. Das Team deckt ein breites Spektrum relevanter klinischer und akademischer Disziplinen ab, darunter die Primärversorgung und spezialisierte Dienste, Epidemiologie, psychische Gesundheit und Gesundheitsökonomie. Das auf 2 Jahre angelegte Programm, in dessen Rahmen 90 neu eingerichtete Long-COVID-Kliniken für groß angelegte Forschungsarbeiten genutzt werden, umfasst 3 wichtige Aspekte.

Zunächst werden der Verlauf und die Inanspruchnahme der Gesundheitsversorgung von Patienten, die in COVID-Kliniken überwiesen wurden, mithilfe von routinemäßigen elektronischen Patientenakten, spezifischen Patientenregistern und Befragungen von Patienten, Gesundheitsfachkräften und politischen Entscheidungsträgern untersucht. Diese Daten werden als Grundlage für politische Maßnahmen dienen, um die Belastung für die Betroffenen und das Gesundheitssystem zu verringern.

Zweitens werden in einer komplexen Interventionsstudie 2 Komponenten eines neuartigen ICP für Long COVID untersucht: Coverscan (ein Multiorgan-MRT-Scan zum Ausschluss von Organschäden) und Living with COVID Recovery (eine digital erweiterte Rehabilitationsplattform). Die Cluster-randomisierte Studie rekrutiert Patienten mit Long COVID, die an spezialisierte Kliniken in 6–10 Gebieten (zunächst Hull, Derby, Leicester, Liverpool, London, Exeter) überwiesen werden. Die Primärversorgungsnetzwerke werden nach dem Zufallsprinzip ausgewählt, um eine Vielfalt an geografischen Bedingungen, klinischen Dienstleistungen und sozioökonomischem Status zu ermöglichen. Darüber hinaus werden in einer eingebetteten Arzneimittel-Plattform-Studie Patienten randomisiert mit Medikamenten behandelt, die für die Behandlung von Long COVID umgewidmet werden könnten: zunächst Rivaroxaban (ein Gerinnungshemmer), Colchicin (ein Entzündungshemmer) und Famotidin/Loratadin (Antihistaminika), basierend auf den vorgeschlagenen zugrunde liegenden Mechanismen, die durch präklinische Daten unterstützt werden, einschließlich Koagulopathie und entzündlicher Zytokinproduktion (Abb 1). Weitere Arzneimittelgruppen werden auf der Grundlage neuer Daten hinzugefügt. Für die Studie sollen 4500 Teilnehmer über einen Zeitraum von 10–12 Monaten rekrutiert werden. Der primäre Endpunkt ist die Erschöpfung auf der Fatigue-Assessment-Skala nach 3 Monaten, aber auch ein breites Spektrum sekundärer Endpunkte (physisch, psychologisch und funktionell) wird erhoben und analysiert.

Drittens werden Ungleichheiten und Stigmatisierung in der Long-COVID-Versorgung untersucht und eine Intervention entwickelt, um die Überweisungen von Mitgliedern solcher Gemeinschaften und Gruppen zu verbessern, die in der derzeitigen Betreuung unterrepräsentiert sind. Darüber hinaus wird eine Mixed-Methods-Studie durchgeführt, um Long COVID und ihre Behandlung mit anderen lang andauernden Krankheiten zu vergleichen und zu kontrastieren, um Informationen für die zukünftige integrierte Behandlung dieser Krankheiten über die Pandemie hinaus zu gewinnen.

Insgesamt hat STIMULATE-ICP das Potenzial, politikrelevante Forschungsergebnisse zu liefern, die von Patienten, Gesundheitsfachkräften und politischen Entscheidungsträgern ergänzt werden, um schnell eine evidenzbasierte, effektive Versorgung für diese neue Erkrankung zu etablieren.

## Schlussfolgerungen

Mit einer ständig wachsenden Patientenpopulation ist Long COVID heute eine weit verbreitete Erkrankung mit sozialen und persönlichen Auswirkungen, die ein besseres Verständnis der Symptomentwicklung, der zugrunde liegenden Mechanismen und der Behandlungen erfordern, um die Gesundheit der Bevölkerung weltweit zu verbessern. Große Fortschritte wurden bei der Entwicklung von Testmodellen erzielt, die das Muster der pathophysiologischen Veränderungen des respiratorischen, autonomen und kardiovaskulären Systems aufzeigen, was die Diagnose erleichtert und die zugrunde liegenden, funktionellen Veränderungen bei Long COVID und verwandten Erkrankungen wie ME/CFS aufdeckt. Den Symptomen kann eine Reihe mechanistischer Veränderungen zugrunde liegen, darunter eine Störung der zellulären Energieproduktion durch mitochondriale Dysfunktion, eine verminderte Sauerstoffversorgung durch Koagulopathie und Endothelschädigung sowie eine Dysregulation des Immunsystems. Rehabilitation kann erfolgreich sein, stellt aber eine Herausforderung dar, wenn es darum geht, eine ausreichende Überwachung zu gewährleisten, um die Aktivität an die physiologischen Fähigkeiten anzupassen und eine Verschlimmerung der Schädigung zu vermeiden. Eine pharmakologische Behandlung ist wahrscheinlich nur bei Untergruppen von Patienten mit bestimmten Symptomen und zugrunde liegender Pathologie wirksam. Daher sind multidisziplinäre Ansätze erforderlich, die Epidemiologie, Immunologie, Multisystemphysiologie und klinische Forschung umfassen, um die verschiedenen interagierenden Prozesse zu verstehen und herauszufinden, wie sie am besten bekämpft werden können, um die Gesundheit wiederherzustellen. Einige dieser Kooperationsprojekte sind bereits im Gange, in Zukunft werden jedoch noch mehr benötigt, um Patienten mit Long COVID zu unterstützen.

## Interessenskonflikt

A.B. ist Treuhänder der South Asian Health Foundation und von Long COVID SOS. B.R. hat von Axcella Therapeutics Honorare für Vorträge erhalten.

## Lizenzangabe

Astin R, Banerjee A, Baker MR, Dani M, Ford E, Hull JH, Lim PB, McNarry M, Morten K, O'Sullivan O, Pretorius E, Raman B, Soteropoulos DS, Taquet M, Hall CN. Long COVID: mechanisms, risk factors and recovery. Exp Physiol. 2023 Jan;108(1):12–27 (DOI: 10.1113/EP090802.©) 2022 The Authors. Experimental Physiology published by John Wiley & Sons Ltd on behalf of The Physiological Society. (Übersetzung; Danksagung, Beiträge der Verfasser gekürzt, Kapitelnummerierung entfernt, Harvard- in Vancouver-Zitierweise geändert), lizensiert unter CC BY 4.0 (https://creativecommons.org/licenses/by/4.0/deed.de).

## Figures and Tables

**Table 1:** Anteil der nach akuter COVID-19 erlittenen Symptome während der Tage 1–180 und 90–180 nach der Erkrankung [13]

Symptom	Anteil der Symptome, %		
	1–180 Tage	90–180 Tage
Angst/Depression	22,82	15,49
Atembeschwerden	18,71	7,94
Abdominale Beschwerden	15,58	8,29
Erschöpfung/Unwohlsein	12,82	5,87
Brust-/Halsschmerzen	12,60	5,71
Sonstige Schmerzen	11,60	7,19
Kopfschmerzen	8,67	4,63
Kognitive Symptome	7,88	3,95
Myalgie	3,24	1,54

## References

[1] Ayoubkhani D, Pawelek P (2022). Prevalence of ongoing symptoms following coronavirus (COVID-19) infection in the UK − Office for National Statistics. https://www.ons.gov.uk/peoplepopulationandcommunity/healthandsocialcare/conditionsanddiseases/%20bulletins/prevalenceofongoingsymptomsfollowingcoronaviruscovid19%20infectionintheuk/6may2022.

[2] Wulf Hanson S, Abbafati C, Aerts J. G, Al-Aly Z, Ashbaugh C, Ballouz T, Blyuss O, Bobkova P, Bonsel G, Borzakova S, Buonsenso D, Butnaru D, Carter A, Chu H, De Rose C, Diab M. M, Ekbom E, El Tantawi M, Fomin V, Vos T (2022). A global systematic analysis of the occurrence, severity, and recovery pattern of long COVID in 2020 and 2021. medRxiv.

[3] Carfì A, Bernabei R, Landi F, Gemelli Against COVID-19 Post-Acute Care Study Group (2020). Persistent symptoms in patients after acute COVID-19. JAMA.

[4] Assaf G, Davis H, McCorkell L, Wei H, O'Neil B, Akrami A, Low R, Mercier J, Adetutu A (2020). What Does COVID-19 Recovery Actually Look Like? − Patient Led Research Collaborative; Covid-19 Body Politic Slack Group. https://patientresearchcovid19.com/esearch/report-1/.

[5] Kirmayer L. J, Groleau D, Looper K. J, Dao M. D (2004). Explaining medically unexplained symptoms. Canadian Journal of Psychiatry − Revue Canadienne De Psychiatrie.

[6] Cabrera Martimbianco A. L, Pacheco R. L, Bagattini Â. M, Riera R (2021). Frequency, signs and symptoms, and criteria adopted for long COVID-19: A systematic review. International Journal of Clinical Practice.

[7] Ziauddeen N, Gurdasani D, O'Hara M. E, Hastie C, Roderick P, Yao G, Alwan N. A (2022). Characteristics and impact of long covid: Findings from an online survey. PLoS One.

[8] Dennis A, Wamil M, Alberts J, Oben J, Cuthbertson D. J, Wootton D, Crooks M, Gabbay M, Brady M, Hishmeh L, Attree E, Heightman M, Banerjee R, Banerjee A, COVERSCAN study investigators. (2021). Multiorgan impairment in low-risk individuals with post-COVID- 19 syndrome: A prospective, community-based study. BMJ Open.

[9] Fernández-de-Las-Peñas C, Palacios-Ceña D, Gómez-Mayordomo V, Florencio L. L, Cuadrado M. L, Plaza-Manzano G, Navarro-Santana M (2021). Prevalence of post-COVID-19 symptoms in hospitalized and non-hospitalized COVID-19 survivors: A systematic review and meta-analysis. European Journal of Internal Medicine.

[10] Sudre C. H, Murray B, Varsavsky T, Graham M. S, Penfold R. S, Bowyer R. C, Pujol J. C, Klaser K, Antonelli M, Canas L. S, Molteni E, Modat M, Jorge Cardoso M, May A, Ganesh S, Davies R, Nguyen L. H, Drew D. A, Astley C. M, Steves C. J (2021). Attributes and predictors of long COVID. Nature Medicine.

[11] Griffanti L, Raman B, Alfaro-Almagro F, Filippini N, Cassar M. P, Sheerin F, Okell T. W, Kennedy McConnell F. A, Chappell M. A, Wang C, Arthofer C, Lange F. J, Andersson J, Mackay C. E, Tunnicliffe E. M, Rowland M, Neubauer S, Miller K. L, Jezzard P, Smith S. M (2021). Adapting the UK biobank brain imaging protocol and analysis pipeline for the C-MORE multi-organ study of COVID-19 survivors. Frontiers in Neurology.

[12] Walker A. J, MacKenna B, Inglesby P, Tomlinson L, Rentsch C. T, Curtis H. J, Morton C. E, Morley J, Mehrkar A, Bacon S, Hickman G, Bates C, Croker R, Evans D, Ward T, Cockburn J, Davy S, Bhaskaran K, Schultze A (2021). Clinical coding of long COVID in English primary care: A federated analysis of 58 million patient records in situ using OpenSAFELY. British Journal of General Practice.

[13] Taquet M, Dercon Q, Luciano S, Geddes J. R, Husain M, Harrison P. J (2021). Incidence, co-occurrence, and evolution of long-COVID features: A 6-month retrospective cohort study of 273,618 survivors of COVID- 19. PLoS Medicine.

[14] Taquet M, Dercon Q, Harrison P. J (2022). Six-month sequelae of post-vaccination SARS-CoV-2 infection: A retrospective cohort study of 10,024 breakthrough infections. Brain, Behavior, and Immunity.

[15] Poenaru S, Abdallah S. J, Corrales-Medina V, Cowan J (2021). COVID-19 and post-infectious myalgic encephalomyelitis/chronic fatigue syndrome: A narrative review. Therapeutic Advances in Infectious Disease.

[16] Singh K. K, Chaubey G, Chen J. Y, Suravajhala P (2020). Decoding SARS-CoV-2 hijacking of host mitochondria in COVID-19 pathogenesis. American Journal of Physiology Cell Physiology.

[17] Tran V-T, Porcher R, Pane I, Ravaud P (2022). Course of post COVID- 19 disease symptoms over time in the ComPaRe long COVID prospective e-cohort. Nature Communication.

[18] Davies K, Dures E, Ng W.-F (2021). Fatigue in inflammatory rheumatic diseases: Current knowledge and areas for future research. Nature Reviews. Rheumatology.

[19] Kluger B. M, Krupp L. B, Enoka R. M (2013). Fatigue and fatigability in neurologic illnesses: Proposal for a unified taxonomy. Neurology.

[20] van Ruitenbeek E, Custers J. A. E, Verhaak C, Snoeck M, Erasmus C. E, Kamsteeg E. J, Schouten M. I, Coleman C, Treves S, Van Engelen B. G, Jungbluth H, Voermans N. C (2019). Functional impairments, fatigue and quality of life in RYR1-related myopathies: A questionnaire study. Neuromuscular Disorders.

[21] Carruthers B. M, van de Sande M. I, De Meirleir K. L, Klimas N. G, Broderick G, Mitchell T, Staines D, Powles A. C. P, Speight N, Vallings R, Bateman L, Baumgarten-Austrheim B, Bell D. S, Carlo-Stella N, Chia J, Darragh A, Jo D, Lewis D, Light A. R, Stevens S (2011). Myalgic encephalomyelitis: International consensus criteria. Journal of Internal Medicine.

[22] Stussman B, Williams A, Snow J, Gavin A, Scott R, Nath A, Walitt B (2020). Characterization of post-exertional malaise in patients with myalgic encephalomyelitis/chronic fatigue syndrome. Frontiers in Neurology.

[23] Komaroff A. L, Fagioli L. R, Doolittle T. H, Gandek B, Gleit M. A, Guerriero R. T, Kornish R. J, Ware N. C, Ware J. E, Bates D. W (1996). Health status in patients with chronic fatigue syndrome and in general population and disease comparison groups. American Journal of Medicine.

[24] Kujawski S, Cossington J, Słomko J, Dawes H, Strong JW, Estevez- Lopez F, Murovska M, Newton JL, Hodges L, Zalewski P (2020). Prediction of discontinuation of structured exercise programme in chronic fatigue syndrome patients. Journal of Clinical Medicine.

[25] Kujawski S, Cossington J, Słomko J, Zawadka-Kunikowska M, Tafil- Klawe M, Klawe J. J, Buszko K, Jakovljevic D. G, Kozakiewicz M, Morten K. J, Dawes H, Strong J. W. L, Murovska M, Van Oosterwijck J, Estevez-Lopez F, Newton J. L, Hodges L, Zalewski P (2021). On Behalf Of The European Network On Me/Cfs Euromene. Relationship between cardiopulmonary, mitochondrial and autonomic nervous system function improvement after an individualised activity programme upon chronic fatigue syndrome patients. Journal of Clinical Medicine.

[26] NICE Guideline [NG206] (2021). Overview | Myalgic encephalomyelitis (or encephalopathy)/chronic fatigue syndrome: Diagnosis and management | Guidance | NICE. https://www.nice.org.uk/guidance/ng206.

[27] Missailidis D, Annesley S. J, Allan C. Y, Sanislav O, Lidbury B. A, Lewis D. P, Fisher P. R (2020). An isolated complex V inefficiency and dysregulated mitochondrial function in immortalized lymphocytes from ME/CFS patients. International Journal of Molecular Sciences.

[28] Sweetman E, Kleffmann T, Edgar C, de Lange M, Vallings R, Tate W (2020). A SWATH-MS analysis of Myalgic Encephalomyelitis/Chronic Fatigue Syndrome peripheral blood mononuclear cell proteomes reveals mitochondrial dysfunction. Journal of Translational Medicine.

[29] Tomas C, Brown A, Strassheim V, Elson J. L, Newton J, Manning P (2017). Cellular bioenergetics is impaired in patients with chronic fatigue syndrome. PLoS One.

[30] Tomas C, Elson J. L, Newton J. L, Walker M (2020). Substrate utilisation of cultured skeletal muscle cells in patients with CFS. Scientific Reports.

[31] Germain A, Giloteaux L, Moore G. E, Levine S. M, Chia J. K, Keller B. A, Stevens J, Franconi C. J, Mao X, Shungu D. C, Grimson A, Hanson M. R (2022). Plasma metabolomics reveals disrupted response and recovery following maximal exercise in myalgic encephalomyelitis/chronic fatigue syndrome. JCI Insight.

[32] Morten K. J, Staines-Urias E, Kenyon J (2018). Potential clinical usefulness of gut microbiome testing in a variety of clinical conditions. Human Microbiome Journal.

[33] Xiong R, Gunter C, Fleming E, Vernon S, Bateman L, Unutmaz D, Oh J (2021). Multi-omics of host-microbiome interactions in short- and long-term Myalgic Encephalomyelitis/Chronic Fatigue Syndrome (ME/CFS). bioRxiv.

[34] Milivojevic M, Che X, Bateman L, Cheng A, Garcia B. A, Hornig M, Huber M, Klimas N. G, Lee B, Lee H, Levine S, Montoya J. G, Peterson D. L, Komaroff A. L, Lipkin W. I (2020). Plasma proteomic profiling suggests an association between antigen driven clonal B cell expansion and ME/CFS. PLoS One.

[35] Phetsouphanh C, Darley DR, Wilson DB, Howe A, Munier CML, Patel SK, Juno JA, Burrell LM, Kent SJ, Dore GJ, Kelleher AD, Matthews GV (2022). Immunological dysfunction persists for 8 months following initial mild-to-moderate SARS-CoV-2 infection. Nature Immunology.

[36] Chang C.-J, Hung L.-Y, Kogelnik A. M, Kaufman D, Aiyar R. S, Chu A. M, Wilhelmy J, Li P, Tannenbaum L, Xiao W, Davis R. W (2021). A comprehensive examination of severely ill ME/CFS patients. Healthcare.

[37] Gaspar-Rodríguez A, Padilla-González A, Rivera-Toledo E (2021). Coronavirus persistence in human respiratory tract and cell culture: An overview. The Brazilian Journal of Infectious Diseases.

[38] Brodin P, Casari G, Townsend L, O'Farrelly C, Tancevski I, Löffler-Ragg J, Mogensen T. H, Casanova J. L, COVID Human Genetic Effort (2022). Studying severe long COVID to understand post-infectious disorders beyond COVID-19. Nature Medicine.

[39] Hamer M, Kivimäki M, Gale C. R, Batty G. D (2020). Lifestyle risk factors, inflammatory mechanisms, and COVID-19 hospitalization: A community-based cohort study of 387,109 adults in UK. Brain, Behavior, and Immunity.

[40] Sallis R, Young D. R, Tartof S. Y, Sallis J. F, Sall J, Li Q, Smith G. N, Cohen D. A (2021). Physical inactivity is associated with a higher risk for severe COVID-19 outcomes: a study in 48 440 adult patients. British Journal of Sports Medicine.

[41] Falkingham BBC K (2020). Long Covid: Oonagh Cousins, GB rower, on the effects − BBC Sport. https://www.bbc.co.uk/sport/rowing/54702500.

[42] Schwellnus M, Sewry N, Snyders C, Kaulback K, Wood P. S, Seocharan I, Derman W, Hull J. H, Valtonen M, Jordaan E (2021). Symptom cluster is associated with prolonged return-to-play in symptomatic athletes with acute respiratory illness (including COVID-19): A cross- sectional study-AWARE study I. British Journal of Sports Medicine.

[43] Holdsworth D. A, Chamley R, Barker-Davies R, O'Sullivan O, Ladlow P, Mitchell J. L, Dewson D, Mills D, May S. L. J, Cranley M, Xie C, Sellon E, Mulae J, Naylor J, Raman B, Talbot N. P, Rider O. J, Bennett A. N, Nicol E. D (2022). Comprehensive clinical assessment identifies specific neurocognitive deficits in working-age patients with long-COVID. PLoS One.

[44] O'Sullivan O, Barker-Davies R, Chamley R, Sellon E, Jenkins D, Burley R, Holden L, Nicol A. M, Phillip R, Bennett A. N, Nicol E, Holdsworth D. A (2021). Defence medical rehabilitation centre (DMRC) COVID-19 recovery service. BMJ Military Health.

[45] O'Sullivan O, Barker-Davies R. M, Thompson K, Bahadur S, Gough M, Lewis S, Martin M, Segalini A, Wallace G, Phillip R, Cranley M (2021). Rehabilitation post-COVID-19: Cross-sectional observations using the Stanford Hall remote assessment tool. BMJ Military Health.

[46] Hull J. H, Wootten M, Moghal M, Heron N, Martin R, Walsted E. S, Biswas A, Loosemore M, Elliott N, Ranson C (2022). Clinical patterns, recovery time and prolonged impact of COVID-19 illness in international athletes: the UK experience. British Journal of Sports Medicine.

[47] Petek B. J, Moulson N, Baggish A. L, Kliethermes S. A, Patel M. R, Churchill T. W, Harmon K. G, Drezner J. A, ORCCA Investigators. (2021). Prevalence and clinical implications of persistent or exertional cardiopulmonary symptoms following SARS-CoV-2 infection in 3597 collegiate athletes: A study from the Outcomes Registry for Cardiac Conditions in Athletes (ORCCA). British Journal of Sports Medicine.

[48] O'Sullivan O (2022). The changing characteristics of post-COVID-19 syndrome: Cross-sectional findings from 458 consultations using the Stanford Hall remote rehabilitation assessment tool. Research Square.

[49] Hull J. H, Wootten M, Ranson C (2022). Tolerability and impact of SARS-CoV-2 vaccination in elite athletes. The Lancet. Respiratory Medicine.

[50] NICE Guideline [NG188] (2020). COVID-19 rapid guideline: managing the long-term effects of COVID-19. National Institute for Health and Care Excellence (NICE).

[51] Barker-Davies R. M, O'Sullivan O, Senaratne K. P. P, Baker P, Cranley M, Dharm-Datta S, Ellis H, Goodall D, Gough M, Lewis S, Norman J, Papadopoulou T, Roscoe D, Sherwood D, Turner P, Walker T, Mistlin A, Phillip R, Nicol A. M, Bahadur S (2020). The Stanford Hall consensus statement for post-COVID-19 rehabilitation. British Journal of Sports Medicine.

[52] O'Sullivan O (2021). Long-term sequelae following previous coronavirus epidemics. Clinical Medicine.

[53] O'Sullivan O, Barker-Davies R, Gough M, Bahadur S, Cranley M, Phillip R (2021). The Stanford Hall coronavirus disease 2019 (COVID-19) remote rehabilitation assessment tool. Future Healthcare Journal.

[54] Wilson M. G, Hull J. H, Rogers J, Pollock N, Dodd M, Haines J, Harris S, Loosemore M, Malhotra A, Pieles G, Shah A, Taylor L, Vyas A, Haddad F. S, Sharma S (2020). Cardiorespiratory considerations for return-to-play in elite athletes after COVID-19 infection: A practical guide for sport and exercise medicine physicians. British Journal of Sports Medicine.

[55] van Hattum J. C, Spies J. L, Verwijs S. M, Verwoert G. C, Planken R. N, Boekholdt S. M, Groenink M, Malekzadeh A, Pinto Y. M, Wilde A. A. M, Jorstad H. T (2021). Cardiac abnormalities in athletes after SARS- CoV-2 infection: A systematic review. BMJ Open Sport & Exercise Medicine.

[56] Ladlow P, O'Sullivan O, Houston A, Barker-Davies R, May S, Mills D, Dewson D, Chamley R, Naylor J, Mulae J, Bennett A. N, Nicol E. D, Holdsworth D. A (2022). Dysautonomia following COVID-19 is not associated with subjective limitations or symptoms but is associated with objective functional limitations. Heart Rhythm.

[57] Fikenzer S, Kogel A, Pietsch C, Lavall D, Stöbe S, Rudolph U, Laufs U, Hepp P, Hagendorff A (2021). SARS-CoV2 infection: Functional and morphological cardiopulmonary changes in elite handball players. Scientific Reports.

[58] Lewis N. A, Collins D, Pedlar C. R, Rogers J. P (2015). Can clinicians and scientists explain and prevent unexplained underperformance syndrome in elite athletes: An interdisciplinary perspective and 2016 update. BMJ Open Sport & Exercise Medicine.

[59] Ladlow P, O'Sullivan O, Bennett A. N, Barker-Davies R, Houston A, Chamley R, May S, Mills D, Dewson D, Rogers-Smith K, Ward C, Taylor J, Mulae J, Naylor J, Nicol E. D, Holdsworth D. A (2022). The effect of medium-term recovery status after COVID-19 illness on cardio-pulmonary exercise capacity in a physically active adult population. Journal of Applied Physiology.

[60] O'Sullivan O, Holdsworth D. A, Ladlow P, Barker-Davies R. M, Chamley R, Houston A, May S, Dewson D, Mills D, Pierce K, Mitchell J, Xie C, Sellon E, Naylor J, Mulae J, Cranley M, Talbot N. P, Rider O. J, Nicol E. D, Bennett A. N (2022). Cardiopulmonary, functional, cognitive and mental health outcomes post COVID, across the range of severity of acute illness, in a physically active working age population. Research Square.

[61] Heightman M, Prashar J, Hillman T. E, Marks M, Livingston R, Ridsdale H. A, Roy K, Bell R, Zandi M, McNamara P, Chauhan A, Denneny E, Astin R, Purcell H, Attree E, Hishmeh L, Prescott G, Evans R, Mehta P, Banerjee A (2021). Post-COVID-19 assessment in a specialist clinical service: A 12-month, single-centre, prospective study in 1325 individuals. BMJ Open Respiratory Research.

[62] Herridge M. S, Cheung A. M, Tansey C. M, Matte-Martyn A, Diaz-Granados N, Al-Saidi F, Cooper A. B, Guest C. B, Mazer C. D, Mehta S, Stewart T. E, Barr A, Cook D, Slutsky A. S, Canadian Critical Care Trials Group (2003). One-year outcomes in survivors of the acute respiratory distress syndrome. New England Journal of Medicine.

[63] Puthucheary Z. A, Rawal J, McPhail M, Connolly B, Ratnayake G, Chan P, Hopkinson N. S, Padhke R, Dew T, Sidhu P. S, Velloso C, Seymour J, Agley C. C, Selby A, Limb M, Edwards L. M, Smith K, Rowlerson A, Rennie M. J, Montgomery H. E (2013). Acute skeletal muscle wasting in critical illness. JAMA.

[64] Rinaldo R. F, Mondoni M, Parazzini E. M, Pitari F, Brambilla E, Luraschi S, Balbi M, Sferrazza Papa G. F, Sotgiu G, Guazzi M, Di Marco F, Centanni S (2021). Deconditioning as main mechanism of impaired exercise response in COVID-19 survivors. European Respiratory Journal.

[65] Baratto C, Caravita S, Faini A, Perego G. B, Senni M, Badano L. P, Parati G (2021). Impact of COVID-19 on exercise pathophysiology: A combined cardiopulmonary and echocardiographic exercise study. Journal of Applied Physiology.

[66] Cassar M. P, Tunnicliffe E. M, Petousi N, Lewandowski A. J, Xie C, Mahmod M, Samat A. H. A, Evans R. A, Brightling C. E, Ho L.-P, Piechnik S. K, Talbot N. P, Holdsworth D, Ferreira V. M, Neubauer S, Raman B (2021). Symptom persistence despite improvement in cardiopulmonary health - insights from longitudinal CMR, CPET and lung function testing post-COVID-19. EClinicalMedicine.

[67] de Boer E, Petrache I, Goldstein N. M, Olin J. T, Keith R. C, Modena B, Mohning M. P, Yunt Z. X, San-Millán I, Swigris J. J (2022). Decreased fatty acid oxidation and altered lactate production during exercise in patients with post-acute COVID-19 syndrome. American Journal of Respiratory and Critical Care Medicine.

[68] Singh I, Joseph P, Heerdt P. M, Cullinan M, Lutchmansingh D. D, Gulati M, Possick J. D, Systrom D. M, Waxman A. B (2022). Persistent exertional intolerance after COVID-19: Insights from invasive cardiopulmonary exercise testing. Chest.

[69] Cortese M, Lee J.-Y, Cerikan B, Neufeldt C. J, Oorschot V. M. J, Köhrer S, Hennies J, Schieber N. L, Ronchi P, Mizzon G, Romero- Brey I, Santarella-Mellwig R, Schorb M, Boermel M, Mocaer K, Beckwith M. S, Templin R. M, Gross V, Pape C, Bartenschlager R (2020). Integrative imaging reveals SARS-CoV-2-induced reshaping of subcellular morphologies. Cell Host & Microbe.

[70] Flynn R. A, Belk J. A, Qi Y, Yasumoto Y, Wei J, Alfajaro M. M, Shi Q, Mumbach M. R, Limaye A, DeWeirdt P. C, Schmitz C. O, Parker K. R, Woo E, Chang H. Y, Horvath T. L, Carette J. E, Bertozzi C. R, Wilen C. B, Satpathy A. T (2021). Discovery and functional inter- rogation of SARS-CoV-2 RNA-host protein interactions. Cell.

[71] Mao L, Jin H, Wang M, Hu Y, Chen S, He Q, Chang J, Hong C, Zhou Y, Wang D, Miao X, Li Y, Hu B (2020). Neurologic manifestations of hospitalized patients with coronavirus disease 2019 in Wuhan, China. JAMA Neurology.

[72] Clavario P, De Marzo V, Lotti R, Barbara C, Porcile A, Russo C, Beccaria F, Bonavia M, Bottaro L. C, Caltabellotta M, Chioni F, Santangelo M, Hautala A. J, Griffo R, Parati G, Corrà U, Porto I (2021). Cardiopulmonary exercise testing in COVID-19 patients at 3 months follow-up. International Journal of Cardiology.

[73] Jimeno-Almazán A, Pallarés J. G, Buendía-Romero Á, Martínez-Cava A, Courel-Ibáñez J (2021). Chronotropic incompetence in non- hospitalized patients with Post-COVID-19 syndrome. Journal of Clinical Medicine.

[74] Abrams R. M. C, Simpson D. M, Navis A, Jette N, Zhou L, Shin S. C (2022). Small fiber neuropathy associated with SARS-CoV-2 infection. Muscle & Nerve.

[75] Blitshteyn S, Whitelaw S (2021). Postural orthostatic tachycardia syndrome (POTS) and other autonomic disorders after COVID-19 infection: A case series of 20 patients. Immunologic Research.

[76] Hinduja A, Moutairou A, Calvet J.-H (2021). Sudomotor dysfunction in patients recovered from COVID-19. Neurophysiologie Clinique = Clinical Neurophysiology.

[77] Johansson M, Ståhlberg M, Runold M, Nygren-Bonnier M, Nilsson J, Olshansky B, Bruchfeld J, Fedorowski A (2021). Long-haul post- COVID-19 symptoms presenting as a variant of postural orthostatic tachycardia syndrome: The Swedish experience. JACC Case Reports.

[78] Shouman K, Vanichkachorn G, Cheshire W. P, Suarez M. D, Shelly S, Lamotte G. J, Sandroni P, Benarroch E. E. S, Berini S. E, Cutsforth- Gregory J. K, Coon E. A, Mauermann M. L, Low P. A, Singer W (2021). Autonomic dysfunction following COVID-19 infection: an early experience. Clinical Autonomic Research.

[79] Sheldon R. S, Grubb B. P, Olshansky B, Shen W.-K, Calkins H, Brignole M, Raj S. R, Krahn A. D, Morillo C. A, Stewart J. M, Sutton R, Sandroni P, Friday K. J, Hachul D. T, Cohen M. I, Lau D. H, Mayuga K. A, Moak J. P, Sandhu R. K, Kanjwal K (2015). 2015 heart rhythm society expert consensus statement on the diagnosis and treatment of postural tachycardia syndrome, inappropriate sinus tachycardia, and vasovagal syncope. Heart Rhythm.

[80] Hausenloy D. J, Arhi C, Chandra N, Franzen-McManus A.-C, Meyer A, Sutton R (2009). Blood pressure oscillations during tilt testing as a predictive marker of vasovagal syncope. Europace.

[81] Julu P. O. O, Cooper V. L, Hansen S, Hainsworth R (2003). Cardio- vascular regulation in the period preceding vasovagal syncope in conscious humans. Journal of Physiology.

[82] Samniah N, Sakaguchi S, Ermis C, Lurie K. G, Benditt D. G (2004). Transient modification of baroreceptor response during tilt-induced vasovagal syncope. Europace.

[83] Buoite Stella A, Furlanis G, Frezza N. A, Valentinotti R, Ajcevic M, Manganotti P (2022). Autonomic dysfunction in post-COVID patients with and witfhout neurological symptoms: A prospective multidomain observational study. Journal of Neurology.

[84] Dantzer R (2018). Neuroimmune interactions: From the brain to the immune system and vice versa. Physiological Reviews.

[85] Baker A. M. E, Maffitt N. J, Vecchio A. D, McKeating K. M, Baker M. R, Baker S. N, Soteropoulos D. S (2022). Neural dysregulation in post- covid fatigue. medRxiv.

[86] Dotan A, David P, Arnheim D, Shoenfeld Y (2022). The autonomic aspects of the post-COVID19 syndrome. Autoimmunity Reviews.

[87] Pan Y, Yu Z, Yuan Y, Han J, Wang Z, Chen H, Wang S, Wang Z, Hu H, Zhou L, Lai Y, Zhou Z, Wang Y, Meng G, Yu L, Jiang H (2021). Alteration of autonomic nervous system is associated with severity and outcomes in patients with COVID-19. Frontiers in Physiology.

[88] Aranow C, Atish-Fregoso Y, Lesser M, Mackay M, Anderson E, Chavan S, Zanos T. P, Datta-Chaudhuri T, Bouton C, Tracey K. J, Diamond B (2021). Transcutaneous auricular vagus nerve stimulation reduces pain and fatigue in patients with systemic lupus erythematosus: A randomised, double-blind, sham-controlled pilot trial. Annals of the Rheumatic Diseases.

[89] Tarn J, Legg S, Mitchell S, Simon B, Ng W-F (2019). The effects of noninvasive vagus nerve stimulation on fatigue and immune responses in patients with primary sjögren's syndrome. Neuromodulation.

[90] Merad M, Blish C. A, Sallusto F, Iwasaki A (2022). The immunology and immunopathology of COVID-19. Science.

[91] Levi M, Thachil J, Iba T, Levy J. H (2020). Coagulation abnormalities and thrombosis in patients with COVID-19. The Lancet Haematology.

[92] Pretorius E, Vlok M, Venter C, Bezuidenhout J. A, Laubscher G. J, Steenkamp J, Kell D. B (2021). Persistent clotting protein pathology in Long COVID/Post-Acute Sequelae of COVID-19 (PASC) is accompanied by increased levels of antiplasmin. Cardiovascular Diabetology.

[93] Willyard C (2020). Coronavirus blood-clot mystery intensifies. Nature.

[94] Zuin M, Engelen M. M, Barco S, Spyropoulos A. C, Vanassche T, Hunt B. J, Vandenbriele C, Verhamme P, Kucher N, Rashidi F, Zuliani G, Konstantinides S. V, Roncon L (2022). Incidence of venous thromboembolic events in COVID-19 patients after hospital discharge: A systematic review and meta-analysis. Thrombosis Research.

[95] Kastenhuber E. R, Mercadante M, Nilsson-Payant B, Johnson J. L, Jaimes J. A, Muecksch F, Weisblum Y, Bram Y, Chandar V, Whittaker G. R, tenOever B. R, Schwartz R. E, Cantley L (2022). Coagulation factors directly cleave SARS-CoV-2 spike and enhance viral entry. eLife.

[96] Al-Aly Z, Bowe B, Xie Y (2022). Long COVID after breakthrough SARS-CoV-2 infection. Nature Medicine.

[97] Xie Y, Xu E, Bowe B, Al-Aly Z (2022). Long-term cardiovascular outcomes of COVID-19. Nature Medicine.

[98] Proal A. D, VanElzakker M. B (2021). Long COVID or post-acute sequelae of COVID-19 (PASC): An overview of biological factors that may contribute to persistent symptoms. Frontiers in Microbiology.

[99] Fogarty H, Townsend L, Morrin H, Ahmad A, Comerford C, Karampini E, Englert H, Byrne M, Bergin C, O'Sullivan J. M, Martin-Loeches I, Nadarajan P, Bannan C, Mallon P. W, Curley G. F, Preston R. J. S, Rehill A. M, McGonagle D, Ni Cheallaigh C (2021). Persistent endotheliopathy in the pathogenesis of long COVID syndrome. Journal of Thrombosis and Haemostasis.

[100] Gavriilaki E, Eftychidis I, Papassotiriou I (2021). Update on endothelial dysfunction in COVID-19: Severe disease, long COVID-19 and pediatric characteristics. Journal of Laboratory Medicine.

[101] Grobler C, Maphumulo S. C, Grobbelaar L. M, Bredenkamp JC, Laubscher G. J, Lourens P. J, Steenkamp J, Kell D. B, Pretorius E (2020). Covid-19: The rollercoaster of fibrin(Ogen), d-dimer, von willebrand factor, p-selectin and their interactions with endothelial cells, platelets and erythrocytes. International Journal of Molecular Sciences.

[102] Bonaventura A, Vecchié A, Dagna L, Martinod K, Dixon D. L, Van Tassell B. W, Dentali F, Montecucco F, Massberg S, Levi M, Abbate A (2021). Endothelial dysfunction and immunothrombosis as key pathogenic mechanisms in COVID-19. Nature Reviews. Immunology.

[103] Althaus K, Marini I, Zlamal J, Pelzl L, Singh A, Häberle H, Mehrländer M, Hammer S, Schulze H, Bitzer M, Malek N, Rath D, Bösmüller H, Nieswandt B, Gawaz M, Bakchoul T, Rosenberger P (2021). Antibody-induced procoagulant platelets in severe COVID-19 infection. Blood.

[104] Fard M. B, Fard S. B, Ramazi S, Atashi A, Eslamifar Z (2021). Thrombosis in COVID-19 infection: Role of platelet activation-mediated immunity. Thrombosis Journal.

[105] Li T, Yang Y, Li Y, Wang Z, Ma F, Luo R, Xu X, Zhou G, Wang J, Niu J, Lv G, Crispe I. N, Tu Z (2022). Platelets mediate inflammatory monocyte activation by SARS-CoV-2 spike protein. Journal of Clinical Investigation.

[106] Grobbelaar L. M, Venter C, Vlok M, Ngoepe M, Laubscher G. J, Lourens P. J, Steenkamp J, Kell D. B, Pretorius E (2021). SARS- CoV-2 spike protein S1 induces fibrin(ogen) resistant to fibrinolysis: Implications for microclot formation in COVID-19. Bioscience Reports.

[107] Kell D. B, Laubscher G. J, Pretorius E (2022). A central role for amyloid fibrin microclots in long COVID/PASC: origins and therapeutic implications. Biochemical Journal.

[108] US Government Accountability Office (2022). Science & Tech Spotlight: Long COVID. GAO-22-105666.

[109] Moriyama M, Nagai M, Maruzuru Y, Koshiba T, Kawaguchi Y, Ichinohe T (2020). Influenza virus-induced oxidized DNA activates inflammasomes. iScience.

[110] Ajaz S, McPhail M. J, Singh K. K, Mujib S, Trovato F. M, Napoli S, Agarwal K (2021). Mitochondrial metabolic manipulation by SARS- CoV-2 in peripheral blood mononuclear cells of patients with COVID-19. American Journal of Physiology Cell Physiology.

[111] Gibellini L, De Biasi S, Paolini A, Borella R, Boraldi F, Mattioli M, LoTartaro D, Fidanza L, Caro-Maldonado A, Meschiari M, Iadisernia V, Bacca E, Riva G, Cicchetti L, Quaglino D, Guaraldi G, Busani S, Girardis M, Mussini C, Cossarizza A (2020). Altered bioenergetics and mitochondrial dysfunction of monocytes in patients with COVID-19 pneumonia. EMBO Molecular Medicine.

[112] Moolamalla S. T. R, Balasubramanian R, Chauhan R, Priyakumar U. D, Vinod P. K (2021). Host metabolic reprogramming in response to SARS- CoV-2 infection: A systems biology approach. Microbial Pathogenesis.

[113] Rinaldo R. F, Mondoni M, Parazzini E. M, Baccelli A, Pitari F, Brambilla E, Luraschi S, Balbi M, Guazzi M, Di Marco F, Centanni S (2021). Severity does not impact on exercise capacity in COVID-19 survivors. Respiratory Medicine.

[114] Evers G, Schulze A. B, Osiaevi I, Harmening K, Vollenberg R, Wiewrodt R, Pistulli R, Boentert M, Tepasse P-R, Sindermann J. R, Yilmaz A, Mohr M (2022). Sustained impairment in cardiopulmonary exercise capacity testing in patients after COVID-19: A single center experience. Canadian Respiratory Journal.

[115] McCully K. K, Natelson B. H, Iotti S, Sisto S, Leigh J. S (1996). Reduced oxidative muscle metabolism in chronic fatigue syndrome. Muscle & Nerve.

[116] Wang L-L, Sun Z, Chen A-P, Wu L-J (2021). 1H NMR-based metabolomics approach for exploring the effect of astaxanthin supplementation on plasma metabolites after high-intensity physical exercise. Journal of Men's Health.

[117] Wong R, Lopaschuk G, Zhu G, Walker D, Catellier D, Burton D, Teo K, Collins-Nakai R, Montague T (1992). Skeletal muscle metabolism in the chronic fatigue syndrome. In vivo assessment by 31P nuclear magnetic resonance spectroscopy. Chest.

[118] Brown A. E, Jones D. E, Walker M, Newton J. L (2015). Abnormalities of AMPK activation and glucose uptake in cultured skeletal muscle cells from individuals with chronic fatigue syndrome. PLoS One.

[119] Fluge Ø, Mella O, Bruland O, Risa K, Dyrstad S. E, Alme K, Rekeland I. G, Sapkota D, Røsland V. G, Fosså A, Ktoridou-Valen I, Lunde S, Sørland K, Lien K, Herder I, Thürmer H, Gotaas M. E, Baranowska K. A, Bohnen L. M. L. J, Tronstad K. J (2017). Metabolic profiling indicates impaired pyruvate dehydrogenase function in myalgic encephalopathy/chronic fatigue syndrome. JCI Insight.

[120] Myhill S, Booth N. E, McLaren-Howard J (2009). Chronic fatigue syndrome and mitochondrial dysfunction. International Journal of Clinical and Experimental Medicine.

[121] Tomas C, Newton J (2018). Metabolic abnormalities in chronic fatigue syndrome/myalgic encephalomyelitis: A mini-review. Biochemical Society Transactions.

[122] Helliwell A. M, Sweetman E. C, Stockwell P. A, Edgar C. D, Chatterjee A, Tate W. P (2020). Changes in DNA methylation profiles of myalgic encephalomyelitis/chronic fatigue syndrome patients reflect systemic dysfunctions. Clinical Epigenetics.

[123] Armstrong C. W, McGregor N. R, Lewis D. P, Butt H. L, Gooley P. R (2015). Metabolic profiling reveals anomalous energy metabolism and oxidative stress pathways in chronic fatigue syndrome patients. Metabolomics.

[124] Naviaux R. K, Naviaux J. C, Li K, Bright A. T, Alaynick W. A, Wang L, Baxter A, Nathan N, Anderson W, Gordon E (2016). Metabolic features of chronic fatigue syndrome. Proceedings National Academy of Science, USA.

[125] Smits B, van den Heuvel L, Knoop H, Küsters B, Janssen A, Borm G, Bleijenberg G, Rodenburg R, van Engelen B (2011). Mitochondrial enzymes discriminate between mitochondrial disorders and chronic fatigue syndrome. Mitochondrion.

[126] Mizuno K, Tanaka M, Nozaki S, Mizuma H, Ataka S, Tahara T, Sugino T, Shirai T, Kajimoto Y, Kuratsune H, Kajimoto O, Watanabe Y (2008). Antifatigue effects of coenzyme Q10 during physical fatigue. Nutrition.

[127] Logan A. C, Wong C (2001). Chronic fatigue syndrome: Oxidative stress and dietary modifications. Alternative Medicine Review.

[128] Castro-Marrero J, Cordero M. D, Segundo M. J, Sáez-Francàs N, Calvo N, Román-Malo L, Aliste L, Fernández de Sevilla T, Alegre J (2015). Does oral coenzyme Q10 plus NADH supplementation improve fatigue and biochemical parameters in chronic fatigue syndrome?. Antioxidants and Redox Signaling.

[129] Castro-Marrero J, Sáez-Francàs N, Segundo M. J, Calvo N, Faro M, Aliste L, Fernández de Sevilla T, Alegre J (2016). Effect of coenzyme Q10 plus nicotinamide adenine dinucleotide supplementation on maximum heart rate after exercise testing in chronic fatigue syndrome − A randomized, controlled, double-blind trial. Clinical Nutrition.

[130] Forsyth L. M, Preuss H. G, MacDowell A. L, Chiazze L, Birkmayer G. D, Bellanti J. A (1999). Therapeutic effects of oral NADH on the symptoms of patients with chronic fatigue syndrome. Annals of Allergy, Asthma & Immunology.

[131] Moriya J, Chen R, Yamakawa J, Sasaki K, Ishigaki Y, Takahashi T (2011). Resveratrol improves hippocampal atrophy in chronic fatigue mice by enhancing neurogenesis and inhibiting apoptosis of granular cells. Biological & pharmaceutical bulletin.

[132] Montoya J, Anderson J, Adolphs D, Bateman L, Klimas N, Levine S, Garvert D, Kaiser J (2018). Original article KPAX002 as a treatment for myalgic encephalomyelitis/chronic fatigue syndrome (ME/CFS): A prospective, randomized trial. International Journal of Clinical and Experimental Medicine.

[133] Poe F. L, Corn J (2020). N-Acetylcysteine: A potential therapeutic agent for SARS-CoV-2. Medical Hypotheses.

[134] Hamill M. J, Daou N, Nitzel A, Pantano L, Koziel M, Chakravarthy M. V (2021). Mechanistic Insights Into AXA1125, a Novel Endogenous Metabolic Modulator Composition, Targeting Multiple NASH Drivers. NASH-TAG.

[135] Shelley J, Hudson J, Mackintosh K. A, Saynor Z. L, Duckers J, Lewis K. E, Davies G. A, Berg R. M. G, McNarry M. A (2021). “I live a kind of shadow life”: Individual experiences of COVID-19 recovery and the impact on physical activity levels. International Journal of Environmental Research and Public Health.

[136] Enright S. J, Unnithan V. B, Heward C, Withnall L, Davies D. H (2006). Effect of high-intensity inspiratory muscle training on lung volumes, diaphragm thickness, and exercise capacity in subjects who are healthy. Physical Therapy.

[137] Beaumont M, Forget P, Couturaud F, Reychler G (2018). Effects of inspiratory muscle training in COPD patients: A systematic review and meta-analysis. The Clinical Respiratory Journal.

[138] McCreery J. L, Mackintosh K. A, Mills-Bennett R, McNarry M. A (2021). The effect of a high-intensity PrO2Fit inspiratory muscle training intervention on physiological and psychological health in adults with bronchiectasis: A mixed-methods study. International Journal of Environmental Research and Public Health.

[139] Severin R, Arena R, Lavie C. J, Bond S, Phillips S. A (2020). Respiratory muscle performance screening for infectious disease management following COVID-19: A highly pressurized situation. American Journal of Medicine.

[140] McNarry M. A, Berg R. M. G, Shelley J, Hudson J, Saynor Z. L, Duckers J, Lewis K, Davies G. A, Mackintosh K. A (2022). Inspiratory muscle training enhances recovery post COVID-19: A randomised controlled trial. European Respiratory Journal.

[141] Campbell H, Hotchkiss R, Bradshaw N, Porteous M (1998). Integrated care pathways. Bmj.

[142] Banerjee A, Heightman M, Murray E, Lorgelly P.N.D STIMULATE- ICP: Understanding long COVID to improve diagnosis, treatment and care. https://www.stimulate-icp.org/about.

[143] Skjørten I, Ankerstjerne O. A. W, Trebinjac D, Brønstad E, Rasch- Halvorsen Ø, Einvik G, Lerum T. V, Stavem K, Edvardsen A, Ingul C. B (2021). Cardiopulmonary exercise capacity and limitations 3 months after COVID-19 hospitalisation. European Respiratory Journal.

